# Comparative Transcriptome Analysis Uncovers the Regulatory Roles of MicroRNAs Involved in Petal Color Change of Pink-Flowered Strawberry

**DOI:** 10.3389/fpls.2022.854508

**Published:** 2022-03-29

**Authors:** Jingyu Yue, Zhixiang Liu, Can Zhao, Jun Zhao, Yang Zheng, Hongwei Zhang, Changhua Tan, Zhentang Zhang, Li Xue, Jiajun Lei

**Affiliations:** ^1^College of Horticulture, Shenyang Agricultural University, Shenyang, China; ^2^College of Biological Science and Technology, Shenyang Agricultural University, Shenyang, China

**Keywords:** pink-flowered strawberry, miRNAs, high-throughput sequencing, flower color, degradome analysis

## Abstract

The pink-flowered strawberry is popular in China due to its high ornamental value. In the present study, sRNAome, transcriptome, and degradome sequencing were performed to understand the functions of microRNAs (miRNAs) and their target genes during flower development in pink-flowered strawberry. Nine small RNA libraries and a mixed degradome library from flower petals at different developmental stages were constructed and sequenced. A total of 739 known miRNAs and 964 novel miRNAs were identified *via* small RNA sequencing, and 639 miRNAs were identified to cleave 2,816 target genes based on the degradome data. Additionally, 317 differentially expressed miRNAs among the various stages of flower development were identified, which regulated 2,134 differentially expressed target genes. These target genes were significantly enriched in the transcriptional regulation, phenylpropanoid biosynthesis, and plant hormone signal transduction pathways. Furthermore, integrated microRNAomic and transcriptomic analyses suggested that 98 miRNAs targeted several transcription factors, including *MYBs* (26), *bHLHs* (12), *NACs* (14), and *SPLs* (19), related to anthocyanin accumulation. In addition, 27 differentially expressed miRNAs might affect anthocyanin biosynthesis by regulating 23 targets involved in the hormone signal transduction pathway. The quantitative real-time PCR (qRT-PCR) analysis confirmed the expression changes of 21 miRNA-target pairs. Furthermore, the transient expression of candidate miRNAs was performed in the pink-flowered strawberry cultivar “Fenyun” at the bud stage. Introduction of FamiR156a, FamiR396e, and FamiR858_R-2 in the “Fenyun” increased flower color intensity, while transient expression of FamiR828a decreased flower color intensity. Overall, the present study uncovers the regulatory functions of microRNAs, including anthocyanin biosynthesis, hormone signaling, and regulation factors during flower development and coloration in pink-flowered strawberry. This work expands the knowledge of miRNAs affecting coloration in strawberry and provides rich resources for future functional studies.

## Introduction

Strawberry (*Fragaria* × *ananassa*), belonging to the Rosaceae family, is one of the most significant horticultural crops grown globally with high economic value. It is rich in anthocyanins which have strong antioxidant and anti-cancer effects (Garcia-Alonso et al., 2005; Speer et al., 2020). The pink-flowered strawberry, an intergeneric hybrid of *Fragaria* and *Potentilla*, was sold as ornamental plants and loved by consumers worldwide due to its colorful petals and fruit production (Xue et al., 2015). Moreover, the plants of pink-flowered strawberry are usually five times costlier than those of white-flowered strawberry because red represents happiness, enthusiasm, and auspiciousness in Chinese traditional culture. Understanding mechanism behind the red petal formation will assist in breeding novel pink-flowered strawberry cultivars.

The petals and fruits of the pink-flowered strawberry are red, but their main anthocyanins are different. The cyanidin-3-glucoside and pelargonidin-3-glucoside are the major anthocyanins in the petals and fruits, respectively (Kosar et al., 2004; da Silva et al., 2007; Xue et al., 2016). Extensive studies have been carried out on the anthocyanin metabolic pathway in strawberry fruits and several genes within the pathway, including *FaCHS* (Hoffmann et al., 2006), *FaCHI* (Deng and Davis, 2001), *FaPAL* (Pombo et al., 2011), *FaF3H*, *FaANR*, *FaANS*, *FaFLS*, *FaLAR* (Almeida et al., 2007; Jiang et al., 2013), *FaUFGT* (Salvatierra et al., 2010), *FaMYB1* (Aharoni et al., 2001), and *FaMYB10* (Lin-Wang et al., 2014) have been identified successively. Transcription factors (TFs) are crucial regulators of anthocyanin accumulation in plants. Among them, MYB/bHLH complexes play a key role. Other TFs, such as *SPL*, *NAC*, *ARF*, and *WRKY* have also been reported to play a regulatory role in anthocyanin biosynthesis (Gou et al., 2011; Qi et al., 2011; Maier et al., 2013; Zhou et al., 2015). Several biochemical and molecular biology studies have explored the mechanism of color formation at the transcription level in pink-flowered strawberry (Xue et al., 2019; Hong et al., 2021). Liu et al. (2021) identified three *R2R3-FaMYB* genes, *FaMYB28*, *FaMYB54*, and *FaMYB576* based on the genome-wide analysis of the *R2R3-MYB* gene family in *F*. × *ananassa*, which regulated anthocyanin biosynthesis in the petal of pink-flowered strawberry. However, the post-transcriptional regulatory role of miRNAs in flower development and color change remains unclear in pink-flowered strawberry.

Plant miRNAs are small non-coding RNAs that are 20–24 nucleotides long and transcribed by non-coding genes. The miRNAs play a pivotal regulatory role in numerous biological processes, including stress response, hormone signal transduction, and metabolite biosynthesis, by negatively regulating key genes at the post-transcriptional stage (Singh and Sharma, 2017; Cai et al., 2019; Das et al., 2021). They are complementary to target mRNAs and can promote the degradation or translation repression of target mRNAs. In the past few decades, many miRNAs have been reported to regulate anthocyanin biosynthesis in plant, such as miR156 (Gou et al., 2011; Liu et al., 2017), miR7125 (Hu et al., 2021), miR828 (Yang et al., 2013; Zhang et al., 2020), miR477 (Dong et al., 2021), and miR858a (Wang et al., 2016). However, studies on the role of miRNAs in regulating strawberry fruit color formation are rare. In order to clarify the post-transcriptional regulation mechanism of flower color formation, flower buds of pink-flowered strawberry cultivar “Sijihong” at three developmental stages were selected to perform a combined analysis of microRNAome, transcriptome, and degradation. These results would provide additional potential targets for the bioengineering and future breeding of new pink-flowered strawberry germplasm.

## Materials and Methods

### Plant Materials and Total RNA Extraction

**Figure 1:**
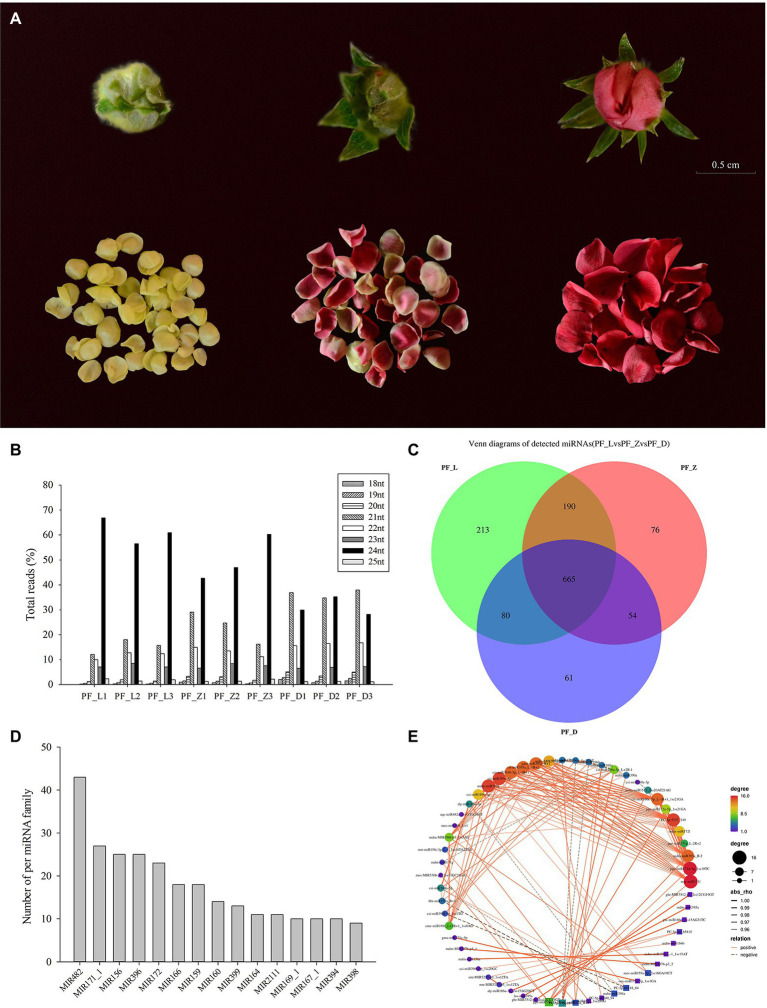
sRNAome data obtained from the different petal developmental stages of pink-flowered strawberry. **(A)** The petal developmental stages of pink-flowered strawberry cultivar Sijihong. **(B)** Length of sRNAs in nine libraries. **(C)** Venn diagram showing the number of differentially expressed microRNAs (miRNAs) during the different petal developmental stages. **(D)** Top 15 known miRNA families. **(E)** The correlation network constructed for 412 miRNAs. The solid lines show a positive correlation, while the dotted lines show a negative correlation. L, young bud stage; Z beginning coloration stage; and D, big bud stage.

The pink-flowered strawberry cultivar “Sijihong” and released by Shenyang Agricultural University in China was used in this study. According to the degree of flower opening and the change in petal color, the developmental stages were categorized into the young bud stage (PF_L), the coloration beginning stage (PF_Z), and the big bud stage (PF_D; Figure 1A; Xue et al., 2019). Petals of these three developmental stages with obviously different colors were collected for miRNA and degradome analyses, with three biological replicates per stage. The pink-flowered strawberry cultivar “Fenyun” was used to perform transient expression of candidate miRNAs and determination of anthocyanin contents. All samples were immediately frozen in liquid nitrogen and stored before −80°C for RNA extraction.

Total RNA was extracted using a modified CTAB method and treated with RNase-free DNase I (Takara, Dalian, China) to remove genomic DNA contamination. The RNA purity was measured by agarose gel (2.0%) electrophoresis. The Nanodrop 2000c spectrophotometer and Agilent 2100 were used to quantify the RNA amount and assess the RNA integrity with RIN number > 7.0, respectively.

### Small RNA Libraries Construction and Bioinformatic Analysis

Approximately, 1 μg of total RNA was used to prepare the sRNA library using the TruSeq Small RNA Sample Preparation Kit (Illumina, United States). Different ends of RNA molecules were ligated to 3′ and 5′ adapters. The ligated RNAs were reverse-transcribed to single-stranded cDNA. The small RNA libraries were used for 50 bp single-end sequencing on an Illumina Hiseq 2500 (LC Bio, China), following the vendor’s recommended protocol. Nine libraries with three biological replicates were constructed from the “PF_L,” “PF_Z,” and “PF_D” samples. The sRNAome sequencing data can be found in NCBI Gene Expression Omnibus (GEO) database (https://www.ncbi.nlm.nih.gov/geo/) under the accession number of GSE193522.

An in-house program was used to remove adapter dimers, junk, less complex, common RNA families, and repeats from the raw reads following the ACGT101-miR pipeline (LC Sciences, Houston, United States). Subsequently, a BLAST search was performed to map the 18–25 nucleotide long unique sequences to species-specific precursors in miRBase 22.0 and identify known miRNAs and novel 3 and 5p miRNAs. The mapped pre-miRNAs were aligned against the reference *Fragaria vesca*^1^ to detect their genomic locations. Then, Bowtie was used to align the unmapped unique sequences, and the RNAfold software was used to predict the hairpin RNA structure in the sequence from the 120 nt flanks. The differentially expressed (DE) miRNAs identified based on the normalized deep sequencing count among the PF_L, PF_Z, and PF_D samples were further analyzed using a Student’s *t*-test and considered statistically significant at *p* < 0.01 and *p* < 0.05.

### Transcriptome Data Analysis

Our transcriptome data (NCBI GEO database under access no. GSE125777) was reanalyzed by mapping to the *F. vesca* genome using the HISAT2 software. Then, StringTie was used to assemble the mapped reads with default parameters. The DE mRNAs with a fold change >2 or <0.5 were selected, and the nested linear models were compared using a parametric F-test (*p* < 0.05) by R package. The heatmap of DE genes was constructed using the TBtools software (Chen et al., 2020).

### Degradome Libraries Construction, *de novo* Assembly, and Bioinformatic Analysis

The poly(A) RNA was obtained from the plant total RNA (20 μg) after two rounds of purification using poly-T oligo-attached magnetic beads. Then, a 3′-adapter random primer was used to reverse the first-strand cDNA. The final cDNA library obtained had an average insert size of 200–400 bp. The degradome library was constructed by mixing samples from three stages and sequenced on the Illumina Hiseq 2500 platform to obtain 50 bp single-end reads.

The original data obtained by degradome sequencing were decoupled and filtered to get the clean data *via* Illumina software. Then, the clean data were compared with the transcriptome data using CleaveLand software, and the matched sequence was recorded to generate the degradation density file. For a given read, only perfectly matched alignments were retained for degradation analysis. Target Finder was used to predict the genes targeted by the most abundant miRNAs (Bai et al., 2020). Further, the miRNA-target regulatory network was analyzed following GO annotation and Kyoto Encyclopedia of Genes and Genomes (KEGG) pathway enrichment analysis using R script.

### Data Validation and Gene Expression Analysis by qRT-PCR

To validate the high-throughput sequencing results, qRT-PCR was performed to examine the DE miRNAs and their targets. The primers of each miRNA and target gene were designed using the miRNA Designed V1.01 and Primer 3.0 software, respectively and listed in Supplementary Table S1. Meanwhile, miRNA first Strand cDNA Synthesis Kit (by stem-loop) and HiScript first Strand cDNA Synthesis Kit (+gDNA wiper; Nazyme, China) were used to transcribe miRNAs and their targets, respectively. Then, the qRT-PCR for miRNAs and their targets were performed using a miRNA Universal SYBR qPCR Master Mix and ChamQ Universal SYBR qPCR Master Mix (Nazyme, China), respectively, on a QuantStudio™ Real-time PCR system (Life, United States) with a final volume of 20 μl. Three biological replicates were used per sample, and the U6 and *FaDBP* (Schaart et al., 2002) genes were used as the reference for miRNAs and target genes, respectively. The petals at young bud stage were used as the control and the relative expression levels were calculated following the 2^-ΔΔCT^ method. Data were analyzed using the multiple range test of Duncan in SPSS v.17.0 and represented as mean ± SE.

### Construction of Transient Expression Vectors of Candidate miRNAs

The precursor sequences of FamiR156a, FamiR396e, FamiR828a, and FamiR858_R-2 were amplified and cloned into the pRI-101 AN (TaKaRa, Dalian, China) binary vector digested by *BamH* I/*Sal* I using the One Step Cloning Kit (Vazyme, China; primers listed in Supplementary Table S1). All constructed vectors were confirmed by sequencing at Sangon Biotech Co., Ltd. (Shanghai, China). Then, those vectors were transformed into *Agrobacterium tumefaciens* (strain GV3101) using the heat shock transformation method.

### Transient Expression and Determination of Anthocyanin Contents in Petals of Pink-Flowered Strawberry

The pink-flowered strawberry cultivar “Fenyun” at the bud stage was infiltrated by *A. tumefaciens* for transient expression. The *Agrobacteria* suspension carrying target constructs was diluted to OD_600_ = 0.5 with suspension buffer (10 mM MES, 10 mM MgCl_2_, and 100 μM acetosyringone) for petal injection. *Agrobacteria* containing pRI-101 AN-FamiR156a, pRI-101 AN-FamiR396e, pRI-101 AN-FamiR858_R-2, and pRI-101 AN-FamiR828a constructs were individually introduced into five “Fenyun” plants, and the empty pRI-101 AN vector as well. All plants were covered with clear plastic lids to maintain the humidity, and kept in dark for 24 h. Plants were then transferred to greenhouse with a 16 h light/8 h dark photoperiod at 25°C for recovery. Petals were collected within 3–7 days after agroinfiltration. The expression of miRNAs and target genes were analyzed by qRT-PCR. The anthocyanin content was measured by the pH differential method (Chen et al., 2015). The sample extracts were diluted 20 times (V: V) in pH 1.0 and pH 4.5 buffer, respectively. After 15 min equilibrium, each solution was measured at 530 and 700 nm by UV–Vis spectrophotometry (UV2450, ShimadzuLtd., Japan). The anthocyanin content was expressed by milligram of cyanidin 3-glucoside equivalents (CGE) mg/g fresh weight (FW).

## Results

### Small RNA Sequencing and Transcriptome Profiling in Petals of Pink-Flowered Strawberry

The pink-flowered strawberry showed a remarkable change in flower color during the petal development, with the deepest flower color at the big bud stage. Small RNA sequencing and transcriptome profiling were performed for different flower developmental stages to explore the expression profiles of miRNA and coding genes, and the molecular changes. After removing low quality sequences and contaminated adaptors, 15,145,282, 9,291,511, and 6,985,639 valid reads were obtained from PF_L, PF_Z, and PF_D, respectively (Supplementary Table S2). The sRNAs showed similar total read length in the nine libraries. The majority of sRNAs were 21 and 24 nt long, with 24 nt ones showing the highest abundance (Figure 1B).

Meanwhile, RNA-seq generated 69.57 Gb of raw reads from nine libraries. After removing low-quality sequences and contaminated adaptors, 47,701,449, 50,216,274, and 54,682,967 valid reads were obtained from PF_L, PF_Z, and PF_D, respectively (Supplementary Table S3). After quality control, the valid reads were mapped to the *F. vesca* reference genome. The mapping ratios of each sample were between 75.31 and 77.41%, with an average value of 76.42%, suggesting genomic difference between pink-flowered strawberry and the *F. vesca* reference genome.

### Known and Novel miRNAs Identified During Petal Coloring of Pink-Flowered Strawberry

A total of 739 known miRNAs and 964 novel miRNAs were identified from the small RNA libraries of pink-flowered strawberry, respectively. Of these mature miRNAs, 213 miRNAs were expressed only in PF_L, 76 in PF_Z, and 61 in PF_D. Meanwhile, 665 miRNAs (348 known and 317 novels) were expressed in all three samples (Figure 1C). These known miRNAs in pink-flowered strawberry could be classified into 85 miRNA families, among which the miR482 family had the largest members of 43, followed by miR171-1 and miR156 families of 27 and 25 members, respectively, while 31 families only identified one member, such as miR481 and miR1862 (Figure 1D).

Among these miRNAs, some miRNAs were highly expressed in PF_L, PF_Z, and PF_D, such as mdm-miR390a and mdm-miR396b, which were accumulated at more than 1,000 TPM. However, 1,056 miRNAs, such as tae-MIR160-p3_1ss5CT and mdm-miR399a_1ss1TG, were expressed at a lower level (TPM < 5). Furthermore, the conserved miRNAs, including those of the same family, exhibited different expression levels. A correlation network was constructed for 412 miRNAs of which expressions were strongly correlated (*R*^2^ ≥ 0.90, *p* < 0.05; Figure 1E). The results showed that most correlations among different miRNAs were positive. And some miRNAs, such as mes-miR171l, csi-miR393c-3p, and csi-miR166c-5p_L-1R + 1_1ss21GA were significantly correlated with more than 10 other miRNAs. There were five novel miRNAs presenting strong and significant correlations, including PC-3p-40_65815, PC-5p-68118_64, PC-3p-5537_549, PC-5p-175_13734, and PC-5p-46040_94.

### Target Predictions of Both Known and Novel miRNAs by Degradome Sequencing

The targets of miRNAs identified in pink-flowered strawberry were predicted *via* degradome sequencing. A total of 23,726,719 raw reads were obtained (Supplementary Table S4). After removing the reads without the adaptors, 23,362,636 reads (98.47%) were successfully mapped to the *F. vesca* genome, and a total of 14,406 targets matched the annotated transcripts in the degradation library *via* Target Finder. CleaveLand identified 2,816 target genes for 639 miRNAs (Supplementary Table S5). Target analysis based on the degradome data showed that more than 80% of miRNAs regulated more than one gene, with the number of target genes varying from 1 to 278. Here, zma-MIR396f-p5_2ss19CT20TC cleaved the largest number of transcripts (278), while 114 miRNAs were cleaved a single target. Meanwhile, a gene could be the target of multiple miRNAs. For example, ath-mir858b-p5 and mdm-miR858 were predicted to regulate MYB82-like genes. The functions of miRNAs were predicted by analyzing the GO and KEGG enrichment of target genes. These analyses showed that miRNAs targeted a wide range of conserved regions in TFs including *MYB*s, *SPL*s, floral homeotic protein, and growth-regulating factor.

### Functions of Differentially Expressed Genes and miRNAs During Petal Coloring of Pink-Flowered Strawberry

**Figure 2:**
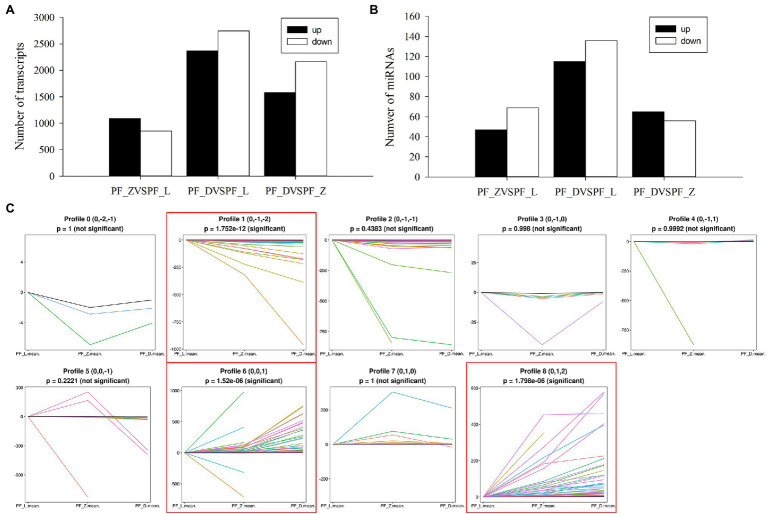
Overview of differentially expressed miRNAs and genes generated *via* high-throughput sequencing of the nine libraries in pink-flowered strawberry. **(A)** The numbers of differentially expressed transcripts in the various comparisons among PF_L, PF_Z, and PF_D. **(B)** The numbers of differentially expressed miRNAs in the various comparisons among PF_L, PF_Z, and PF_D. **(C)** Expression patterns of differentially expressed miRNAs during flower development from profile 0 to 8. Only profiles 1, 6, and 8 showed significantly different trends ( *p* < 0.05).

To better understand the dynamic flower development, the DE genes and miRNAs in the nine libraries of pink-flowered strawberry were analyzed. As shown in Figure 2A, more DE genes were detected in PF_L vs. PF_D with 2,368 upregulated genes and 2,746 downregulated genes. Meanwhile, 1,940 and 3,741 DE genes were detected in PF_L vs. PF_Z and PF_D vs. PF_Z, respectively. To further analyze the biological functions of DE genes in pink-flowered strawberry, KEGG enrichment analysis was performed in PF_L vs. PF_Z, PF_L vs. PF_D, PF_Z vs. PF_D, and PF_L vs. PF_Z vs. PF_D. The DE genes were significantly enriched in plant hormone signal transduction and primary and secondary metabolic pathways, such as biosynthesis of amino acids, glutathione metabolism, terpenoid backbone biosynthesis, and phenylpropanoid biosynthesis (Supplementary Figure S1). Notably, 118 DE genes related to phenylpropanoid biosynthesis were identified in PF_L vs. PF_D, probably involved in petal color change during flower development. Meanwhile, the plant hormone signal transduction was significantly enriched in each stage of flower development.

Meanwhile, 251, 121, and 116 DE miRNAs were identified in PF_L vs. PF_D, PF_D vs. PF_Z, and PF_L vs. PF_Z, respectively (Figure 2B). According to the changes in the expression levels, these DE miRNAs could be categorized into nine groups (profiles 0–8; Figure 2C), among which profile 1, profile 6, and profile 8 showed significantly difference (*p* < 0.05). The profile 1 was continuously upregulated including 63 miRNAs, while profiles 6 and 8 were downregulated, including 75 and 65 miRNAs, respectively. A total of 2,134 DE genes targeted by DE miRNAs were identified in pink-flowered strawberry. These target genes were involved in transcriptional regulation, plant hormone signal transduction, phenylpropanoid biosynthesis, spliceosome, and endocytosis (Supplementary Figure S2). Collectively, these results confirm the significant role of miRNAs in petal color formation of pink-flowered strawberry.

### Identification and Characterization of Genes and miRNAs Related to Petal Color Change of Pink-Flowered Strawberry

**Figure 3:**
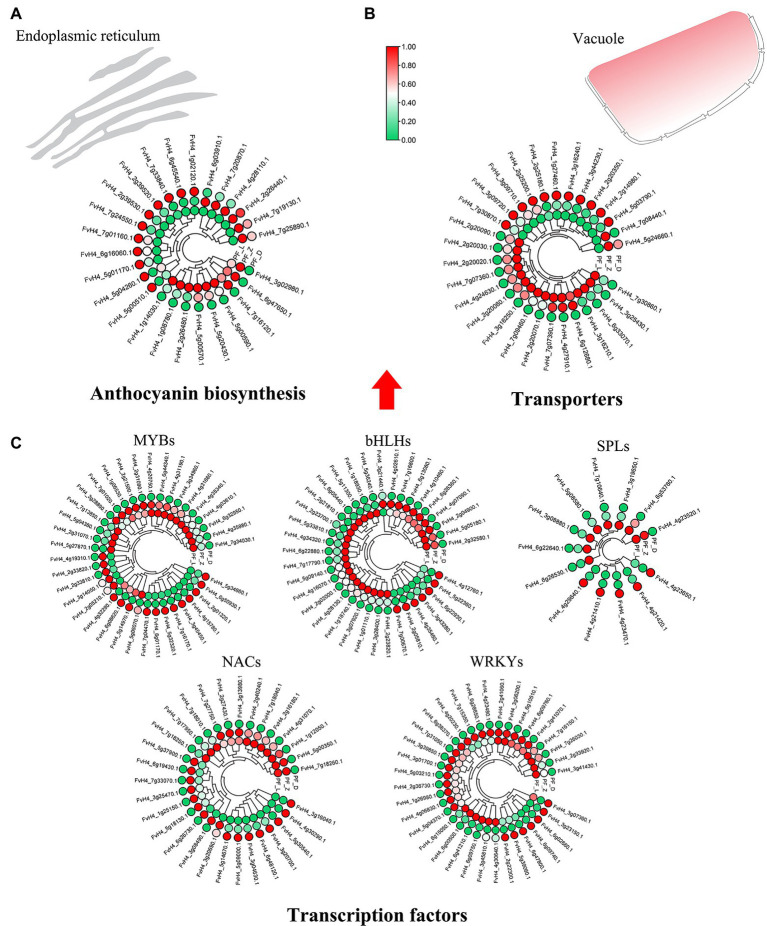
Heatmaps showing the expression patterns of the differentially expressed genes involved in anthocyanin biosynthesis. **(A)** Differentially expressed structural genes of the anthocyanin biosynthesis pathway. **(B)** Differentially expressed transporters involved in anthocyanin biosynthesis. **(C)** Differentially expressed transcription factors, including *MYBs*, *bHLHs*, *SPLs*, *NACs*, and *WRKYs*, which regulate anthocyanin biosynthesis.

The key genes involved in flower color formation of pink-flowered strawberry were summarized from the transcriptome database. The annotations of 56 DE genes were related the anthocyanin biosynthesis, including anthocyanin and flavonol synthesis-related structural genes, anthocyanin modified genes, and anthocyanin transporters (Supplementary Table S6). Majority of DE structural genes showed a gradual increase in expression during flower development (Figure 3A). Two phenylalanine ammonia-lyases (PALs) phenylpropanoid biosynthesis pathway were DE. Among other DE genes, one chalcone synthase (CHS), two chalcone isomerases (CHI), four flavanone 3-hydroxylases (F3H), three flavonoid 3′-hydroxylases (F3’H), and one flavonoid 3′,5′-hydroxylase (F3’5’H) were DE during the upstream of anthocyanin biosynthesis, and three DE dihydroflavonol 4-reductases (DFR), two DE anthocyanidins synthases (ANS), and four DE UDP-glycosyltransferases (UGTs) were in the downstream of anthocyanin biosynthesis. Three DE genes *FvCHS* (FvH4_7g01160), *FvDFR* (FvH4_2g39520), and *FvANS* (FvH4_5g01170), significantly increased in PF_L vs. PF_D. In addition, two types of anthocyanin transporters were obtained. There were 16 DE genes encoding glutathione S—transferases F (GSTF) and 13 ABCC transporter family members, such as *FvGSTF* (FvH4_1g27460, FvH4_3g16240) and *FvABCC* genes (FvH4_3g44230) showing significant difference in PF_L vs. PF_D (log_2_ fold change > 3; Figure 3B). Several TFs have been confirmed to play an important role in the petal color change. In this study, 173 DE TFs, such as MYBs (37 genes), bHLHs (36 genes), SPLs (13 genes), and NACs (30 genes), were identified in the PF_L vs. PF_Z vs. PF_D. The expression patterns of the DE TFs in the three developmental stages were shown in Figure 3C. Among the DE TFs, the majority exhibited a lower expression in PF_D than the control PF_L stage.

**Figure 4:**
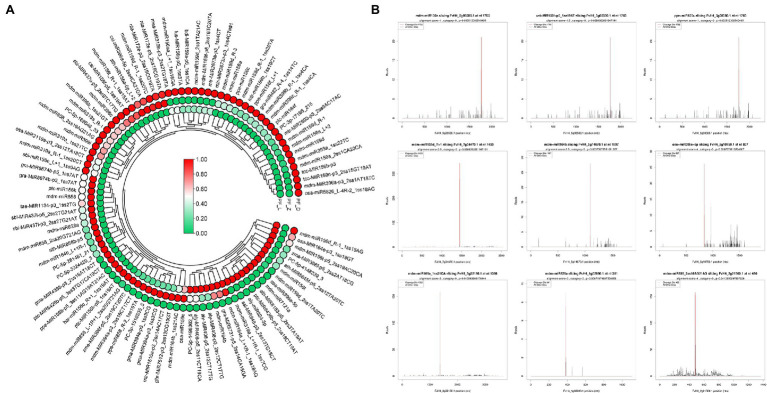
Differentially expressed miRNAs involved in anthocyanin biosynthesis and target (T) plots of miRNAs verified by degradome sequencing. **(A)** Heatmaps showing the expression patterns of the differentially expressed miRNAs involved in anthocyanin biosynthesis. **(B)** The plots of the targets cleaved by the corresponding miRNAs from the degradome library. The red line represents the predicted cleavage sites of the corresponding miRNAs.

Although no anthocyanin synthesis-related structural genes were regulated by DE miRNAs, many TFs potentially regulating anthocyanin biosynthesis were targeted by 98 DE miRNAs (Figure 4), of which 26 targeted *FvMYBs* (Supplementary Figure S3A), 12 targeted *FvbHLHs* (Supplementary Figure S3B), 14 targeted *FvNACs* (Supplementary Figure S3C), and 19 targeted *FvSPLs* (Supplementary Figure S3D). There were eight targeted *FvMYBs*, including *FvMYB4*, *FvMYB5*, *FvMYB6-1*, *FvMYB6-2*, *FvMYB82*, *FvMYB114*, *FvMYB308*, and *FvGAMYB*. As shown in Supplementary Figure S3A, five miRNA-targets showed negative correlations, such as mdm-miR828a (target *FvMYB114*) and mdm-miR858 (target *FvMYB6*). Most miRNA-*FvMYB*s showed a reverse expression pattern only in one development stage. For example, ppe-miR858_R-2 showed first increased at PF_Z and then decreased at PF_D, while its target *FvMYB308* (FvH4_2g01320.1) showed continuous accumulation during flower development. There were seven targeted *FvbHLHs*, including *FvbHLH30*, *FvbHLH77*, *FvbHLH79*, *FvbHLH100*, *FvbHLH113*, *FvbHLH137*, and *FvbHLH157*, in which six miRNA-targets showed reverse expression patterns, such as osa-miR396-5p (target *FvbHLH79*); There were six targeted *FvSPLs*, including *FvSPL9*, *FvSPL13A*, *FvSPL6*, *FvSPL12*, and *FvSPL16*. Most miRNA-*FvSPL*s showed reverse expression patterns. For example, the expression levels of mdm-miR156a increased during development, while its target (*FvSPL13A*) decreased. These results suggested that the flower development and petal color change in pink-flowered strawberry might be primarily regulated by miRNAs, through control of TF expression.

**Figure 5:**
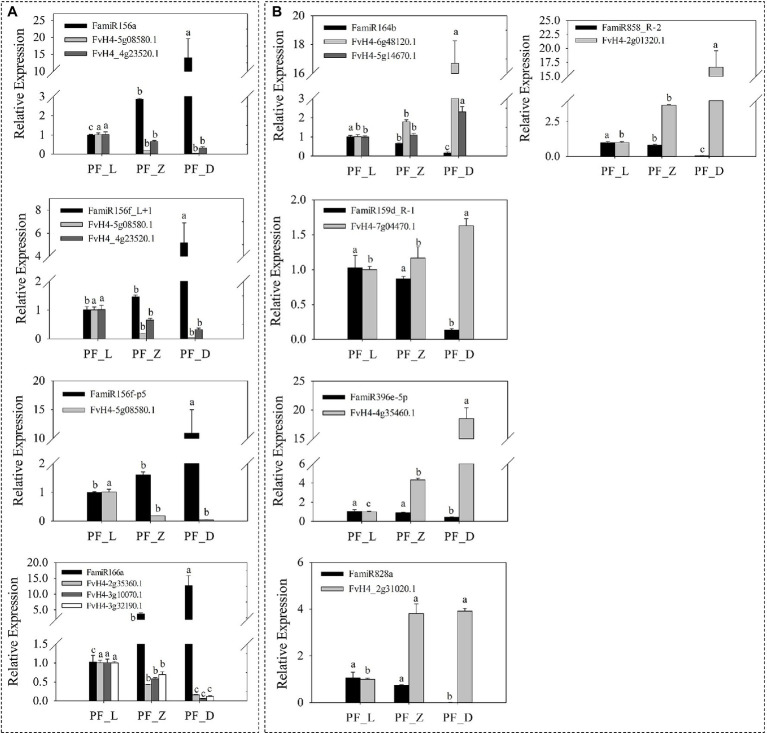
Expression patterns of nine anthocyanin-related miRNAs and corresponding candidate targets in pink-flowered strawberry. **(A)** The expressions of miRNAs showed an upward trend, while their corresponding targets showed a downward trend during flower development. **(B)** The expression levels of miRNAs decreased gradually, while the expression levels of corresponding targets increased gradually. L, young bud stage; Z, beginning coloration stage; and D, big bud stage. Error bars show the SEs from three biological replicates and the lowercase indicates statistical significance at *p* < 0.05.

Furthermore, nine miRNAs and corresponding candidate targets were selected for qRT-PCR analysis to validate the expression patterns of miRNAs and their targets. Our results supported downregulation of target genes mediated by miRNAs (Figure 5). With the development of flower and the deepening of petal color, the expression levels of the four miRNAs, including FamiR156a (targeting *FaSPL13* FvH4_5g08580.1 and *FaSPL16* FvH4_4g23520.1), FamiR156f-L + 1 (targeting *FaSPL9* FvH4_6g22640.1 and *FaSPL13* FvH4_5g08580.1), and FamiR156f-P5 (targeting *FaSPL13* FvH4_5g08580.1) gradually increased while the expression levels of their corresponding targets gradually decreased (Figure 5A). The other five miRNAs including FamiR164b (targeting *FaNAC* FvH4_5g14670.1 and FvH4_6g48120.1), FamiR159d-R-1 (targeting *FaGAMYB* FvH4_7g04470.1), FamiR396e (targeting *FabHLH79* FvH4_4g35460.1), FamiR828a (targeting *FaMYB114* FvH4_2g31020.1), and FamiR858_R-2 (targeting *FaMYB308* FvH4_2g01320.1), showed an opposite trend of which their expression levels significantly decreased, while their target genes significantly increased (Figure 5B).

### Screening of Target Genes and miRNAs Related to Hormone Signal Transduction Pathways

According to the KEGG analysis, eight signal transduction pathways, including auxin, cytokinin, gibberellins, and abscisic acid, were annotated in the transcriptome data of pink-flowered strawberry. A total of 177 DE genes were identified, among which 38 were involved in auxin signal transduction, 15 in cytokinin signal transduction, three in gibberellin signal transduction, 36 in abscisic acid signal transduction, 32 in ethylene signal transduction, 27 in brassinosteroid signal transduction, 20 in jasmonate signal transduction, and six in salicylic acid signal transduction (Supplementary Figure S4). In the auxin signaling pathway, six DE genes encoding auxin transporters (Aux1), nine encoding auxin-responsive proteins IAA (AUX1/IAA), 13 encoding auxin response factors (ARF), and seven encoding auxin-responsive GH3 showed distinct expression dynamics across three developmental stages. Multiple *FvARF* genes, were highly expressed in PF_L, and gradually decreased alone the development of flower; However, two *FvAUX1* genes (FvH4_6g11840, FvH4_2g22520) showed high fold change between PF_L and PF_D (>3.5), and their expression levels increased gradually alone flower development. Most DE genes within the cytokinin pathway were expressed higher in PF_L than PF_D, such as one *FvAHP* gene (FvH4_3g01870) and two *FvARR* genes (FvH4_7g25970, FvH4_1g01300). Their expression level decreased gradually during petal development.

Three DE genes were involved in gibberellin signaling pathway, in which one transcript encoding *FvGID* (FvH4_6g04960) was differentially expressed during flower development. A total of 29 transcripts of probable protein phosphatase 2C gene (PP2C) were annotated in abscisic acid signaling pathway, most of which showed increased expression levels; FvH4_7g31810 showed a large fold change of 4.17 between PF_L and PF_D. As shown in Supplementary Figure S4, most of the DE genes of the ethylene signal transduction pathway were expressed higher in PF_L than PF_D. Additionally, seven and 14 transcripts were annotated as serine/threonine-protein kinases (CTR) and ethylene-responsive TFs (ERF), respectively. Three transcripts FvH4_2g26080, FvH4_7g26410, and FvH4_4g03470 encoding *FvERF* genes showed high accumulation at PF_L. Seven transcripts encoding serine/threonine-protein kinase (BSK), 10 encoding xyloglucan endotransglucosylase/hydrolase (TCH4), and four encoding cyclin-D3 (CYCD3) involving in the brassinosteroid signal transduction were identified. The expression level of the *FvTCH4* gene (FvH4_4g22090) decreased first and then increased during development, corroborated with its FPKM value. In addition, the expression level of the *JAR1* gene (FvH4_7g16040) within the jasmonic acid pathway increased gradually and reached the maximum at the PF_D stage.

**Figure 6:**
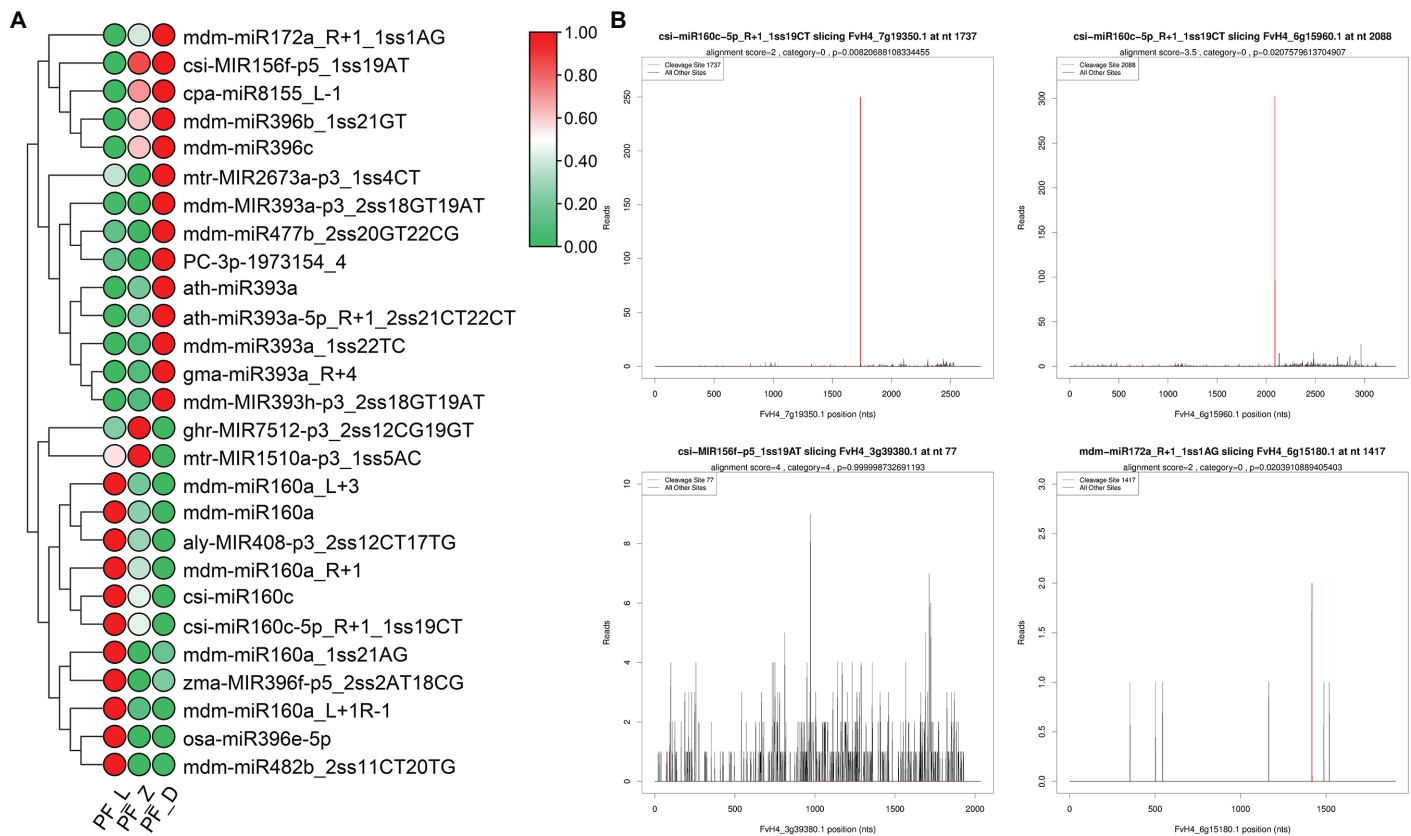
Differently expressed miRNAs involved in hormone signal transduction pathway and target (T) plots of miRNAs verified by degradome sequencing. **(A)** Heatmaps showing expression patterns of the different expression miRNAs involved in hormone signal transduction pathway. **(B)** T plots of the targets cleaved by the corresponding miRNAs from the degradome library. The redline represents the predicted cleavage site of the corresponding miRNAs.

We further investigated the target genes of miRNAs involved in plant hormone signal transduction based on the degradome data. Twenty-seven DE miRNAs participated in auxin, GA, ethylene, jasmonate, and abscisic acid signal transduction (Figure 6). Meanwhile, 14 miRNAs targeting eight genes of auxin signal transduction accounted for half of the miRNAs related to hormone signal transduction pathway. Four *ARF* genes were targeted by seven miR160s, including mdm-miR160a, mdm-miR160a_R + 1, mdm-miR160a_L + 3, mdm-miR160a_L + 1R-1, mdm-miR160a_1ss21AG, csi-miR160c, and csi-miR160c-5p_R + 1_1ss19CT (TPM > 50). From bud stage to full bloom stage, the expression of mdm-miR160a, mdm-miR160a_L + 1R-1, mdm-miR160a_R + 1, and csi-miR160c-5p_R + 1_1ss19CT gradually decreased; in contrast, the expression level of the candidate target genes gradually increased. Different miR393 members targeted *FaAUX1* and *FaTIR1* (Supplementary Figure S5). In the abscisic acid signal transduction pathway, six miRNAs targeted six transcripts of *FvPP2C* gene family. In ethylene signal transduction, three miRNAs (mdm-miR172a_R + 1_1ss1AG, csi-MIR156f-p5_1ss19AT, and zma-MIR396f-p5_2ss2AT18CG) targeted *FvERF* and *FvCTR1*. Additionally, mdm-miR172a_R + 1_1ss1AG and csi-MIR156f-p5_1ss19AT showed a progressive increase of expression during flower development, leading to reduction of *FvERF* expression. On the contrary, zma-MIR396f-p5_2ss2AT18CG showed relatively high expression levels during the initial developmental stages, while its target *FvCTR1* showed an opposite expression pattern (Supplementary Figure S5). Our data suggest miRNAs could alter the expression of key genes related to auxin, GA, and ABA pathways during flower development.

**Figure 7:**
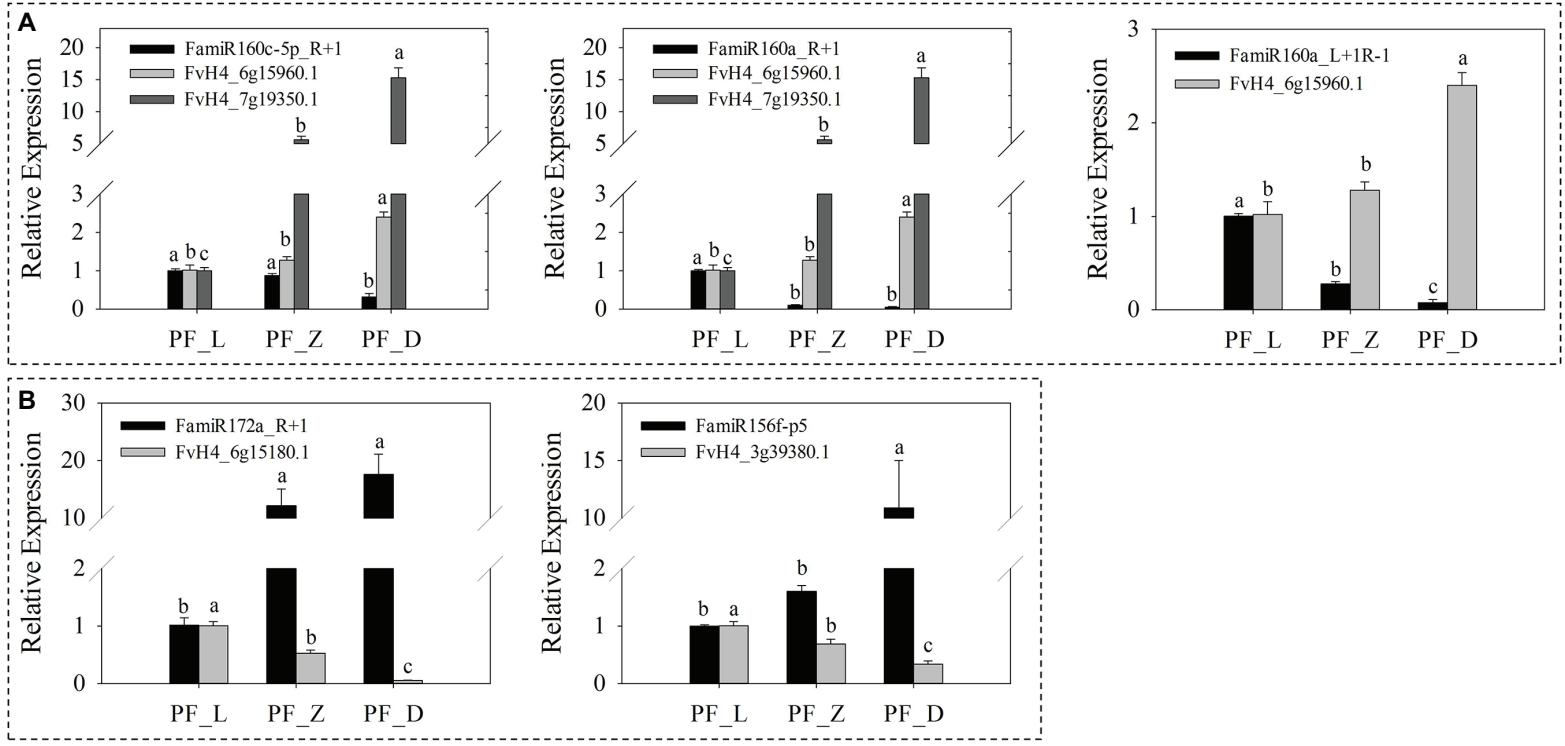
Expression patterns of five miRNAs and corresponding candidate targets involved in plant hormone signal transduction in pink-flowered strawberry. **(A)** The expression levels of miRNAs decreased gradually, while their expression levels of corresponding targets increased gradually. **(B)** The expressions of miRNAs showed an upward trend, while the corresponding target genes showed a downward trend during flower development. L, young bud stage; Z, beginning coloration stage; and D, big bud stage. Error bars show the SEs from three biological replicates and the lowercase indicates statistical significance at *p* < 0.05.

Furthermore, five identified miRNAs were selected and their expression was measured by the qRT-PCR. Along flower development, a progressive decrease in FamiR160a_L + 1R-1 was observed, while the abundance of its target *FaARF* (FvH4_6g15960.1) increased significantly. Similar miRNA target expression patterns were also found for FamiR160c-5p_R + 1 and FamiR160a_R + 1 (Figure 7A). Indeed, for these FamiR160 members, a significant decrease (*p* < 0.05) in abundance was seen in PF_L vs. PF_D, and their target *FaARF* (FvH4_7g19350.1) showed a progressive increase (*p* < 0.05). The FamiR172a_R + 1 was found highly accumulated at PF_D, opposite to the target *FaERF* (FvH4_6g15180.1) gene. The pair of FamiR156f-p5 and its target *FaCTR1* (FvH4_3g39380.1) exhibited the same trend (Figure 7B).

### Functions of Candidate miRNAs Involved in Anthocyanin Metabolism

**Figure 8:**
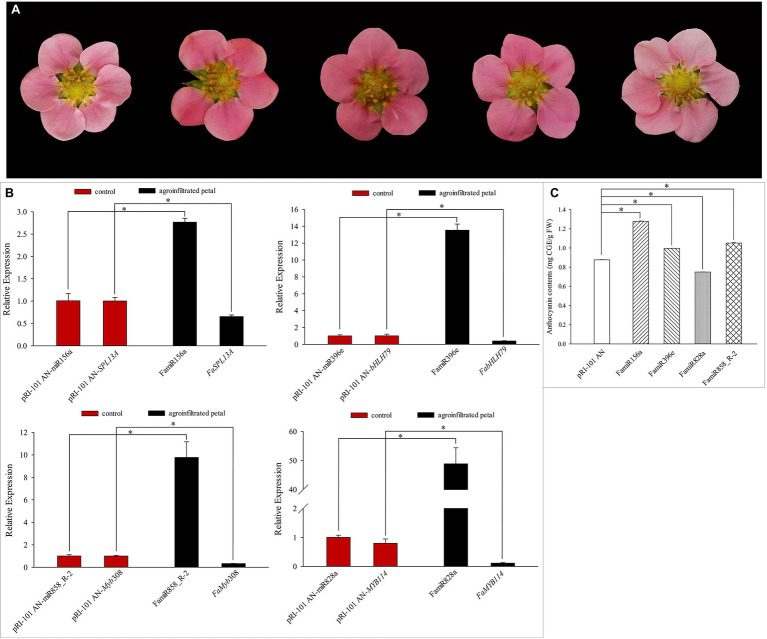
Transient expression of candidate miRNAs in pink-flowered strawberry cultivar “Fenyun.” **(A)** Petal color after injection. From left to right: the pRI-101 AN (control), pRI-101 AN-FamiR156a, pRI-101 AN-FamiR396e, pRI-101 AN-FamiR858_R-2, and pRI-101 AN-FamiR828a. **(B)** Expression levels of candidate miRNAs and targets after injection. **(C)** The anthocyanin contents in petals after injecting candidate miRNAs. The symbol * indicates statistical significance at *p* < 0.05.

We analyzed transient expression of petal and the functions of candidate miRNAs involved in coloration in pink-flowered strawberry “Fenyun.” The petals injected with pRI-101 AN were used as the control. We observed changes of flower color phenotypes within 3–7 days after injection (Figure 8A). Compared with the control, the petal color turned darker after injecting pRI-101 AN-FamiR156a, pRI-101 AN-FamiR396e, and pRI-101 AN-FamiR858_R-2, while the petal color turned lighter after injecting pRI-101 AN-FamiR828a. Expression levels of candidate miRNAs and corresponding target genes were determined by qRT-PCR. The result showed that expression levels of overexpressed candidate miRNAs were significantly higher than those of the control, while the expression levels of their corresponding target genes were decreased (Figure 8B). The anthocyanin contents in agroinfiltrated petals of pRI-101 AN-FamiR156a, pRI-101 AN-FamiR396e, and pRI-101 AN-FamiR858_R-2 were significantly more than those in the control (Figure 8C), indicating that transient expression of these miRNAs activated the anthocyanin accumulation, while the content in pRI-101 AN-FamiR828a was less than that in the control, indicating FamiR828a inhibited the anthocyanin accumulation in “Fenyun.”

## Discussion

The present study provides the first comprehensive overview of miRNAs expression dynamics during flower development and coloring in pink-flowered strawberry. High throughput sequencing technology provided us with an efficient method to identify miRNAs. A large number of miRNAs have been identified in many plants, including tomato (Karlova et al., 2013), grapevine (Pantaleo et al., 2010), and sweet potato (He et al., 2019; Tang et al., 2020). Up to date, many *Fragaria* miRNAs have been identified and deposited to miRBase 22.0 (Ge et al., 2013; Li et al., 2019). Xu et al. (2013) obtained 88 known miRNAs and 1,244 new candidate miRNAs involved in fruit senescence of *F.* × *ananassa*. In this study, a total of 739 known miRNAs and 964 novel miRNAs were identified in PF_L, PF_Z and PF_D libraries, expanding the strawberry miRNA database, and facilitate the understanding of the regulatory mechanisms underlying coloration in pink-flowered strawberries.

Both structural genes encoding anthocyanin biosynthesis-related enzymes and TFs controlling the expression of structural genes regulate anthocyanin synthesis. In this study, 56 DE structural genes and 173 TFs, such as *MYB* (37 genes), *bHLH* (36 genes), *NAC* (30 genes), and *SPL* families (13 genes), were identified during flower development. No miRNAs were found to regulate structural genes related to anthocyanin biosynthesis directly. However, miRNA families of FamiR156s, FamiR159s, FamiR396s, FamiR828s, and FamiR858s potentially targeted *FvSPLs*, *FvMYBs*, and *FvbHLHs*. The *MYB* genes have been identified as one of the major regulators of anthocyanin biosynthesis pathway. Four subgroups of *R2R3-MYBs* related to the flavonoid synthesis have been identified in *Arabidopsis thaliana*, including subgroup 4, subgroup 5, subgroup 6, and subgroup 7 (Dubos et al., 2010). In this study, according to degradome data and expression profiles of DE miRNA-targets, a total of eight *FvMYBs* were targeted by different miRNAs. Through constructing phylogenetic tree with *AtR2R3-MYBs* in *A. thaliana*, *FvMYB308* and *FvGAMYB* were found to belong to subgroup 4, which inhibited anthocyanin synthesis, *FvMYB114* in subgroup 6 promoted anthocyanin synthesis, and *FvMYB4* and *FvMYB5* in subgroup 7 controlled the synthesis of flavonoids in petals of pink-flowered strawberry (Supplementary Figure S6A). Therefore, those corresponding miRNAs might have a regulatory function in petal coloring of pink-flowered strawberry including FamiR828a (target *FvMYB114* FvH4_2g31020.1), FamiR159d-R-1 (target *FvGAMYB* FvH4_7g04470.1), and FamiR858_R-2 (target *FvMYB308* FvH4_2g01320.1).

Previous studies indicated that *AtMYB114*, *PyMYB114*, and *MdMYB114* were active regulators of anthocyanin biosynthesis in *Arabidopsis* (Gonzalez et al., 2008), pear (Yao et al., 2017), and apple (Jiang et al., 2021). Other studies suggested that *MdMYB12* and *MrMYB6* negatively regulate anthocyanin accumulation in apple (Mao et al., 2021) and Chinese bayberry (Shi et al., 2021). Here, our degradome analysis showed that FamiRNA828a and FamiRNA858_R-2 targeted *FaMYB114* and *FaMYB308*, respectively. The expression of FamiR828a was opposite to that of *FvMYB114*, suggesting a negative correlation between FamiR828a and anthocyanin accumulation in the petal of pink-flowered strawberry. Similarity, FamiR858_R-2 negatively regulated *FaMYB308*, which might effectively influence the target genes involved in anthocyanin biosynthesis in the petal of pink-flowered strawberry. In addition, a previous study demonstrated that miR828 and miR858 promoted anthocyanin and flavanol accumulation in grape by regulating *VvMYB114*. And in apple, miR858 and miR828 interacted with multiple *MYBs* to form a complex regulatory network involved in regulating anthocyanin metabolism (Xia et al., 2012; Guan et al., 2014). Therefore, FamiR828a and FamiR858_R-2 identified in this study might play an important role in anthocyanin biosynthesis in the petal of pink-flowered strawberry.

The regulatory role of several *AtbHLH*s related to anthocyanin synthesis has been identified in *Arabidopsis*, such as *AtbHLH1*, *AtbHLH2*, and *AtbHLH42* (Heim et al., 2003). It was found five *FvbHLHs* (*FvbHLH30*, *FvbHLH77*, *FvbHLH79*, *FvbHLH113*, and *FvbHLH137*) may relate to the anthocyanin synthesis *via* phylogenetic analysis with *AtbHLH*s (Supplementary Figure S6B). Their corresponding miRNAs might affect the petal coloration of pink-flowered strawberry including zma-mir396f-p5 (targeting *FvbHLH30*), mdm-miR393a (targeting *FvbHLH77*), mdm-miR396b (targeting *FvbHLH79*), nta-mir172e-p3 (targeting *FvbHLH113*), and osa-miR5826_L-4R-2 (targeting *FvbHLH137*). Xia et al. (2012) performed deep smallRNA-seq in apple and showed that MdTAS4-siR81(−) targeted a *bHLH* TF to regulate anthocyanin biosynthesis. MicroRNA396 has been demonstrated to regulate flower development (Liu et al., 2009; Omidbakhshfard et al., 2015). Yuan et al. (2020) reported that miR396 overexpression affected anthocyanin synthesis and led to green spikelets in regenerated transgenic creeping bentgrass. In our study, FamiR396e-5p cleaved *FabHLH* in degradome sequencing. We found that the expression of *FvbHLH* increased, while that of FamiR396e-5p decreased during flower development. These observations indicated the important roles of FamiR396e-5p and *FvbHLH* gene in regulating anthocyanin accumulation.

Previous studies have demonstrated that miR156s positively regulate anthocyanin biosynthesis by targeting *SPL* TFs, and *SPLs* negatively regulate anthocyanin accumulation through MYB-bHLH-WD40 complex (Gou et al., 2011). Wang et al. (2020) reported that three microRNA-target modules, including miR156-*SPL*s, miR160h-*ARF18*, and miR858-*MYB39*, may control anthocyanin accumulation in miR156 overexpressed transgenic poplar. In our study, the expression levels of FamiR156s gradually increased during flower development, in contrast to their target genes. These results suggest that FamiR156s-*SPL*s may be involved in anthocyanin accumulation. Hormones are important factors inducing anthocyanin accumulation. The ABA, ethylene, and jasmonate promote the accumulation of anthocyanin, while auxin inhibits anthocyanin accumulation (Loreti et al., 2008; Gao et al., 2021). Wang et al. (2018) reported that *MdARF13* as a repressor directly bound to the promoter of *MdDFR* to regulate anthocyanin biosynthesis in apple. Based on these reports, we hypothesize that some growth regulators might promote flower coloration, and affect anthocyanin accumulation. In our study, *FvARF* was downregulated while *FvERF* was upregulated during flower development, and their corresponding miRNAs showed a reverse trend, suggesting the role of miR160s and miR172s in regulating anthocyanin accumulation through the auxin and ethylene signaling pathways. These data collectively supported that miRNAs could alter the expression of key genes related to hormone biosynthesis and were involved in the regulation of flower development and coloring in pink-flowered strawberry.

## Conclusion

Our comprehensive analysis of the sRNAome, transcriptome, and degradome indicated the regulatory functions of microRNAs in flower development and color changes of pink-flowered strawberry. We analyzed the changes in miRNAs expression level and predicted the target genes of the candidate miRNAs. Our analysis revealed that 98 miRNAs targeted several TFs, including *MYBs*, *bHLHs*, and *SPLs*, related to anthocyanin accumulation. Additionally, 27 DE miRNAs may affect anthocyanin metabolism by regulating 23 genes in the hormone signal transduction pathway. Given the key role of plant microRNAs in various processes, further genetic and molecular evidence based on loss of function and overexpression analyses is needed to verity miRNA functions and its regulatory role in anthocyanin biosynthesis in petal. These results expand the pool of identified miRNAs in strawberry, which may serve as important resources for future research and also provides a foundation for using miRNAs to change strawberry flower color.

## Data Availability Statement

The datasets presented in this study can be found in online repositories. The names of the repository/repositories and accession number(s) can be found in the article/Supplementary Material.

## Author Contributions

JY and LX conceived the study and drafted the manuscript. ZL and CZ analyzed the sRNAsome data. JZ and YZ analyzed the transcriptome data. HZ, CT, and ZZ analyzed the degradome data. JL revised the manuscript and provided guidance on the whole study. All authors contributed to the article and approved the submitted version.

## Funding

This study was supported by the National Natural Science Foundation of China (31701964).

## Conflict of Interest

The authors declare that the research was conducted in the absence of any commercial or financial relationships that could be construed as a potential conflict of interest.

## Publisher’s Note

All claims expressed in this article are solely those of the authors and do not necessarily represent those of their affiliated organizations, or those of the publisher, the editors and the reviewers. Any product that may be evaluated in this article, or claim that may be made by its manufacturer, is not guaranteed or endorsed by the publisher.

## Footnotes

^1^ https://www.rosaceae.org/species/fragaria_vesca/genome_v4.0.a2

## References

[ref1] Aharoni A. De Vos C. R. Wein M. Sun Z. Greco R. Kroon A.. (2001). The strawberry *FaMYB1* transcription factor suppresses anthocyanin and flavonol accumulation in transgenic tobacco. Plant J. 28, 319–332. doi: 10.1046/j.1365-313X.2001.01154.x, PMID: 11722774

[ref2] Almeida J. R. M. D’Amico E. Preuss A. Carbone F. de Vos C. R. Deiml B.. (2007). Characterization of major enzymes and genes involved in flavonoid and proanthocyanidin biosynthesis during fruit development in strawberry (*Fragaria* × *ananassa*). Arch. Biochem. Biophys. 465, 61–71. doi: 10.1016/j.abb.2007.04.040, PMID: 17573033

[ref3] Bai S. Tian Y. Tan C. Bai S. Hao J. Hasi A. (2020). Genome-wide identification of microRNAs involved in the regulation of fruit ripening and climacteric stages in melon (*Cucumis melo*). Hortic. Res. 7, 1–13. doi: 10.1038/s41438-020-0331-3 32637134 PMC7327070

[ref4] Cai H. Yang C. X. Liu S. Qi H. R. Wu L. Xu L. A.. (2019). MiRNA-target pairs regulate adventitious rooting in *Populus*: a functional role for miR167a and its target auxin response factor 8. Tree Physiol. 39, 1922–1936. doi: 10.1093/treephys/tpz085, PMID: 31504994

[ref5] Chen C. J. Chen H. Zhang Y. Thomas H. R. Frank M. H. He Y. H.. (2020). TBtools: an integrative toolkit developed for interactive analyses of big biological data. Mol. Plant 13, 1194–1202. doi: 10.1016/j.molp.2020.06.009, PMID: 32585190

[ref6] Chen S. Zhang F. Ning J. Liu X. Zhang Z. Yang S. (2015). Predicting the anthocyanin content of wine grapes by NIR hyperspectral imaging. Food Chem. 172, 788–793. doi: 10.1016/j.foodchem.2014.09.119, PMID: 25442621

[ref7] da Silva F. L. Escribano-Bailón M. T. Alonso J. J. P. Rivas-Gonzalo J. C. Santos-Buelga C. (2007). Anthocyanin pigments in strawberry. LWT Food Sci. Technol. 40, 374–382. doi: 10.1016/j.lwt.2005.09.018, PMID: 34471319

[ref8] Das R. Mukherjee A. Basak S. Kundu P. (2021). Plant miRNA responses under temperature stress. Plant Gene 28:100317. doi: 10.1016/j.plgene.2021.100317, PMID: 34747296

[ref9] Deng C. Davis T. M. (2001). Molecular identification of the yellow fruit color (c) locus in diploid strawberry: a candidate gene approach. Theor. Appl. Genet. 103, 316–322. doi: 10.1007/s001220100648

[ref10] Dong X. N. Liu C. R. Wang Y. Q. Dong Q. Gai Y. P. Ji X. L. (2021). MicroRNA profiling during mulberry (*Morus atropurpurea* Roxb) fruit development and regulatory pathway of miR477 for anthocyanin accumulation. Front. Plant Sci. 12:687364. doi: 10.3389/fpls.2021.687364, PMID: 34567022 PMC8455890

[ref11] Dubos C. Stracke R. Grotewold E. Weisshaar B. Martin C. Lepiniec L. (2010). MYB transcription factors in *Arabidopsis*. Trends Plant Sci. 15, 573–581. doi: 10.1016/j.tplants.2010.06.005, PMID: 20674465

[ref12] Gao H. N. Jiang H. Cui J. Y. You C. X. Li Y. Y. (2021). The effects of hormones and environmental factors on anthocyanin biosynthesis in apple. Plant Sci. 312:111024. doi: 10.1016/j.plantsci.2021.111024, PMID: 34620429

[ref13] Garcia-Alonso M. Rimbach G. Sasai M. Nakahara M. Matsugo S. Uchida Y.. (2005). Electron spin resonance spectroscopy studies on the free radical scavenging activity of wine anthocyanins and pyranoanthocyanins. Mol. Nutr. Food Res. 49, 1112–1119. doi: 10.1002/mnfr.200500100, PMID: 16254886

[ref14] Ge A. J. Shangguan L. F. Zhang X. Dong Q. H. Han J. Liu H.. (2013). Deep sequencing discovery of novel and conserved microRNAs in strawberry (*Fragaria* × *ananassa*). Physiol. Plant. 148, 387–396. doi: 10.1111/j.1399-3054.2012.01713.x, PMID: 23061771

[ref15] Gonzalez A. Zhao M. Z. Leavitt J. M. Lloyd A. M. (2008). Regulation of the anthocyanin biosynthetic pathway by the TTG1/bHLH/Myb transcriptional complex in *Arabidopsis* seedlings. Plant J. 53, 814–827. doi: 10.1111/j.1365-313X.2007.03373.x, PMID: 18036197

[ref16] Gou J. Y. Felippes F. F. Liu C. J. Weigel D. Wang J. W. (2011). Negative regulation of anthocyanin biosynthesis in *Arabidopsis* by a miR156-targeted SPL transcription factor. Plant Cell 23, 1512–1522. doi: 10.1105/tpc.111.084525, PMID: 21487097 PMC3101539

[ref17] Guan X. Y. Pang M. X. Nah G. Shi X. L. Ye W. X. Stelly D. M.. (2014). miR828 and miR858 regulate homoeologous *MYB2* gene functions in *Arabidopsis* trichome and cotton fibre development. Nat. Commun. 5:3050. doi: 10.1038/ncomms4050, PMID: 24430011

[ref18] He L. H. Tang R. M. Shi X. W. Wang W. B. Cao Q. H. Liu X. Y.. (2019). Uncovering anthocyanin biosynthesis related microRNAs and their target genes by small RNA and degradome sequencing in tuberous roots of sweet potato. BMC Plant Biol. 19:232. doi: 10.1186/s12870-019-1790-2, PMID: 31159725 PMC6547535

[ref19] Heim M. A. Jakoby M. Werber M. Martin C. Weisshaar B. Bailey P. C. (2003). The basic helix-loop-helix transcription factor family in plants: a genome-wide study of protein structure and functional diversity. Mol. Biol. Evol. 20, 735–747. doi: 10.1093/molbev/msg088, PMID: 12679534

[ref20] Hoffmann T. Kalinowski G. Schwab W. (2006). RNAi-induced silencing of gene expression in strawberry fruit (*Fragaria* × *ananassa*) by agroinfiltration: a rapid assay for gene function analysis. Plant J. 48, 818–826. doi: 10.1111/j.1365-313X.2006.02913.x, PMID: 17092319

[ref21] Hong Y. H. Ye Q. H. Li Z. K. Wang W. Xie Q. Chen Q. X.. (2021). Accumulation of anthocyanins in red-flowered strawberry ‘Meihong’ petals and expression analysis of *MYB* gene. Acta Hortic. Sinica 48, 1470–1484. doi: 10.16420/j.issn.0513-353x.2020-0477

[ref22] Hu Y. J. Cheng H. Zhang Y. Zhang J. Niu S. Q. Wang X. S.. (2021). The MdMYB16/MdMYB1-miR7125-MdCCR module regulates the homeostasis between anthocyanin and lignin biosynthesis during light induction in apple. New Phytol. 231, 1105–1122. doi: 10.1111/nph.17431, PMID: 33908060

[ref23] Jiang S. H. Sun Q. G. Zhang T. L. Liu W. J. Wang N. Chen X.. (2021). *MdMYB114* regulates anthocyanin biosynthesis and functions downstream of MdbZIP4-like in apple fruit. J. Plant Physiol. 257:153353. doi: 10.1016/j.jplph.2020.153353, PMID: 33352460

[ref24] Jiang F. Wang J. Y. Jia H. F. Jia W. S. Wang H. Q. Xiao M. (2013). RNAi-mediated silencing of the flavanone 3-hydroxylase gene and its effect on flavonoid biosynthesis in strawberry fruit. J. Plant Growth Regul. 32, 182–190. doi: 10.1007/s00344-012-9289-1

[ref25] Karlova R. van Haarst J. C. Maliepaard C. van de Geest H. Bovy A. G. Lammers M.. (2013). Identification of microRNA targets in tomato fruit development using high-throughput sequencing and degradome analysis. J. Exp. Bot. 64, 1863–1878. doi: 10.1093/jxb/ert049, PMID: 23487304 PMC3638818

[ref26] Kosar M. Kafkas E. Paydas S. Baser K. H. C. (2004). Phenolic composition of strawberry genotypes at different maturation stages. J. Agric. Food Chem. 52, 1586–1589. doi: 10.1021/jf035093t, PMID: 15030215

[ref27] Li Y. P. Pi M. T. Gao Q. Liu Z. C. Kang C. Y. (2019). Updated annotation of the wild strawberry *Fragaria vesca* V4 genome. Hortic. Res. 6:61. doi: 10.1038/s41438-019-0142-6, PMID: 31069085 PMC6491553

[ref28] Lin-Wang K. McGhie T. K. Wang M. Liu Y. Warren B. Storey R.. (2014). Engineering the anthocyanin regulatory complex of strawberry (*Fragaria vesca*). Front. Plant Sci. 5:651. doi: 10.3389/fpls.2014.00651, PMID: 25477896 PMC4237049

[ref29] Liu R. Lai B. Hu B. Qin Y. H. Hu G. B. Zhao J. T. (2017). Identification of microRNAs and their target genes related to the accumulation of anthocyanins in *Litchi chinensis* by high-throughput sequencing and degradome analysis. Front. Plant Sci. 7:2059. doi: 10.3389/fpls.2016.02059, PMID: 28119728 PMC5223483

[ref30] Liu H. N. Shu Q. Lin-Wang K. Allan A. C. Espley R. V. Su J.. (2021). The PyPIF5-*PymiR156a*-PySPL9-PyMYB114/MYB10 module regulates light-induced anthocyanin biosynthesis in red pear. Mol. Hortic. 1, 1–14. doi: 10.1186/s43897-021-00018-5 PMC10514999 37789406

[ref31] Liu D. M. Song Y. Chen Z. X. Yu D. Q. (2009). Ectopic expression of miR396 suppresses *GRF* target gene expression and alters leaf growth in *Arabidopsis*. Physiol. Plant. 136, 223–236. doi: 10.1111/j.1399-3054.2009.01229.x, PMID: 19453503

[ref32] Loreti E. Povero G. Novi G. Solfanelli C. Alpi A. Perata P. (2008). Gibberellins, jasmonate and abscisic acid modulate the sucrose-induced expression of anthocyanin biosynthetic genes in *Arabidopsis*. New Phytol. 179, 1004–1016. doi: 10.1111/j.1469-8137.2008.02511.x, PMID: 18537890

[ref33] Maier A. Schrader A. Kokkelink L. Falke C. Welter B. Iniesto E.. (2013). Light and the E3 ubiquitin ligase COP1/SPA control the protein stability of the MYB transcription factors PAP1 and PAP2 involved in anthocyanin accumulation in *Arabidopsis*. Plant J. 74, 638–651. doi: 10.1111/tpj.12153, PMID: 23425305

[ref34] Mao Z. L. Jiang H. Y. Wang S. Wang Y. C. Yu L. Zou Q.. (2021). The MdHY5-MdWRKY41-MdMYB transcription factor cascade regulates the anthocyanin and proanthocyanidin biosynthesis in red-fleshed apple. Plant Sci. 306:110848. doi: 10.1016/j.plantsci.2021.110848, PMID: 33775373

[ref35] Omidbakhshfard M. A. Proost S. Fujikura U. Mueller-Roeber B. (2015). Growth regulating factors (GRFs): a small transcription factor family with important functions in plant biology. Mol. Plant 8, 998–1010. doi: 10.1016/j.molp.2015.01.013, PMID: 25620770

[ref36] Pantaleo V. Szittya G. Moxon S. Miozzi L. Moulton V. Dalmay T.. (2010). Identification of grapevine microRNAs and their targets using high-throughput sequencing and degradome analysis. Plant J. 62, 960–976. doi: 10.1111/j.0960-7412.2010.04208.x, PMID: 20230504

[ref37] Pombo M. A. Martínez G. A. Civello P. M. (2011). Cloning of *FaPAL6* gene from strawberry fruit and characterization of its expression and enzymatic activity in two cultivars with different anthocyanin accumulation. Plant Sci. 181, 111–118. doi: 10.1016/j.plantsci.2011.04.012, PMID: 21683875

[ref38] Qi T. C. Song S. S. Ren Q. C. Wu D. W. Huang H. Chen Y.. (2011). The jasmonate-ZIM-domain proteins interact with the WD repeat/bHLH/MYB complexes to regulate jasmonate-mediated anthocyanin accumulation and trichome initiation in *Arabidopsis thaliana*. Plant Cell 23, 1795–1814. doi: 10.1105/tpc.111.083261, PMID: 21551388 PMC3123955

[ref39] Salvatierra A. Pimentel P. Moya-Leon M. A. Caligari P. D. Herrera R. (2010). Comparison of transcriptional profiles of flavonoid genes and anthocyanin contents during fruit development of two botanical forms of *Fragaria chiloensis* ssp. *chiloensis*. Phytochemistry 71, 1839–1847. doi: 10.1016/j.phytochem.2010.08.005, PMID: 20800857

[ref40] Schaart J. Salentijn E. Krens F. (2002). Tissue-specific expression of the β-glucuronidase reporter gene in transgenic strawberry (*Fragaria* × *ananassa*) plants. Plant Cell Rep. 21, 313–319. doi: 10.1007/s00299-002-0514-4

[ref41] Shi L. Y. Chen X. Wang K. Yang M. J. Chen W. Yang Z. F.. (2021). *MrMYB6* from Chinese bayberry (*Myrica rubra*) negatively regulates anthocyanin and proanthocyanidin accumulation. Front. Plant Sci. 12:685654. doi: 10.3389/fpls.2021.685654, PMID: 34220906 PMC8253226

[ref42] Singh N. Sharma A. (2017). Turmeric (*Curcuma longa*): miRNAs and their regulating targets are involved in development and secondary metabolite pathways. C. R. Biol. 340, 481–491. doi: 10.1016/j.crvi.2017.09.009, PMID: 29126713

[ref43] Speer H. D’Cunha N. M. Alexopoulos N. I. McKune A. J. Naumovski N. (2020). Anthocyanins and human health—a focus on oxidative stress, inflammation and disease. Antioxidants 9:366. doi: 10.3390/antiox9050366, PMID: 32353990 PMC7278778

[ref44] Tang C. Han R. P. Zhou Z. K. Yang Y. Y. Zhu M. K. Xu T.. (2020). Identification of candidate miRNAs related in storage root development of sweet potato by high throughput sequencing. J. Plant Physiol. 251:153224. doi: 10.1016/j.jplph.2020.153224, PMID: 32634748

[ref45] Wang K. Li T. T. Chen S. Q. Rashid A. (2020). The biochemical and molecular mechanisms of softening inhibition by chitosan coating in strawberry fruit (*Fragaria* × *ananassa*) during cold storage. Sci. Hortic. 271:109483. doi: 10.1016/j.scienta.2020.109483

[ref46] Wang Y. L. Wang Y. Q. Song Z. Q. Zhang H. Y. (2016). Repression of *MYBL2* by both microRNA858a and HY5 leads to the activation of anthocyanin biosynthetic pathway in *Arabidopsis*. Mol. Plant 9, 1395–1405. doi: 10.1016/j.molp.2016.07.003, PMID: 27450422

[ref47] Wang Y. C. Wang N. Xu H. F. Jiang S. H. Fang H. C. Su M. Y.. (2018). Auxin regulates anthocyanin biosynthesis through the aux/IAA-ARF signaling pathway in apple. Hortic. Res. 5:59. doi: 10.1038/s41438-018-0068-4, PMID: 30534386 PMC6269505

[ref48] Xia R. Zhu H. An Y. Q. Beers E. P. Liu Z. R. (2012). Apple miRNAs and tasiRNAs with novel regulatory networks. Genome Biol. 13:R47. doi: 10.1186/gb-2012-13-6-r47, PMID: 22704043 PMC3446319

[ref49] Xu X. B. Yin L. L. Ying Q. C. Song H. M. Xue D. W. Lai T. F.. (2013). High-throughput sequencing and degradome analysis identify miRNAs and their targets involved in fruit senescence of *Fragaria ananassa*. PLoS One 8:e70959. doi: 10.1371/journal.pone.0070959, PMID: 23990918 PMC3747199

[ref50] Xue L. Dai H. P. Lei J. J. (2015). Creating high polyploidy pink-flowered strawberries with improved cold tolerance. Euphytica 206, 417–426. doi: 10.1007/s10681-015-1499-8

[ref51] Xue L. Wang Z. G. Zhang W. Li Y. X. Wang J. Lei J. J. (2016). Flower pigment inheritance and anthocyanin characterization of hybrids from pink-flowered and white-flowered strawberry. Sci. Hortic. 200, 143–150. doi: 10.1016/j.scienta.2016.01.020

[ref52] Xue L. Wang J. Zhao J. Zheng Y. Wang H. F. Wu X.. (2019). Study on cyanidin metabolism in petals of pink-flowered strawberry based on transcriptome sequencing and metabolite analysis. BMC Plant Biol. 19:423. doi: 10.1186/s12870-019-2048-8, PMID: 31610785 PMC6791029

[ref53] Yang F. X. Cai J. Yang Y. Liu Z. B. (2013). Overexpression of microRNA828 reduces anthocyanin accumulation in *Arabidopsis*. Plant Cell Tissue Organ Cult. 115, 159–167. doi: 10.1007/s11240-013-0349-4

[ref54] Yao G. F. Ming M. L. Allan A. C. Gu C. Li L. T. Wu X.. (2017). Map-based cloning of the pear gene *MYB114* identifies an interaction with other transcription factors to coordinately regulate fruit anthocyanin biosynthesis. Plant J. 92, 437–451. doi: 10.1111/tpj.13666, PMID: 28845529

[ref55] Yuan S. R. Li Z. G. Yuan N. Hu Q. Zhou M. Zhao J. M.. (2020). MiR396 is involved in plant response to vernalization and flower development in *Agrostis stolonifera*. Hortic. Res. 7:173. doi: 10.1038/s41438-020-00394-x, PMID: 33328434 PMC7603517

[ref56] Zhang B. Yang H. J. Yang Y. Z. Zhu Z. Z. Li Y. N. Qu D.. (2020). Mdm-miR828 participates in the feedback loop to regulate anthocyanin accumulation in apple peel. Front. Plant Sci. 11:608109. doi: 10.3389/fpls.2020.608109, PMID: 33391322 PMC7774908

[ref57] Zhou H. Wang K. L. Wang H. L. Gu C. Dare A. P. Espley R. V.. (2015). Molecular genetics of blood-fleshed peach reveals activation of anthocyanin biosynthesis by NAC transcription factors. Plant J. 82, 105–121. doi: 10.1111/tpj.12792, PMID: 25688923

