# Supplementary material for: Comparative Transcriptome Analysis Uncovers the Regulatory Roles of MicroRNAs Involved in Petal Color Change of Pink-Flowered Strawberry
Source: Front Plant Sci. 2022 Mar 29;13:854508. doi: 10.3389/fpls.2022.854508 (PMC9002178; doi:10.3389/fpls.2022.854508)
Supplement: Supplementary file 1 [file Data_Sheet_1.PDF]

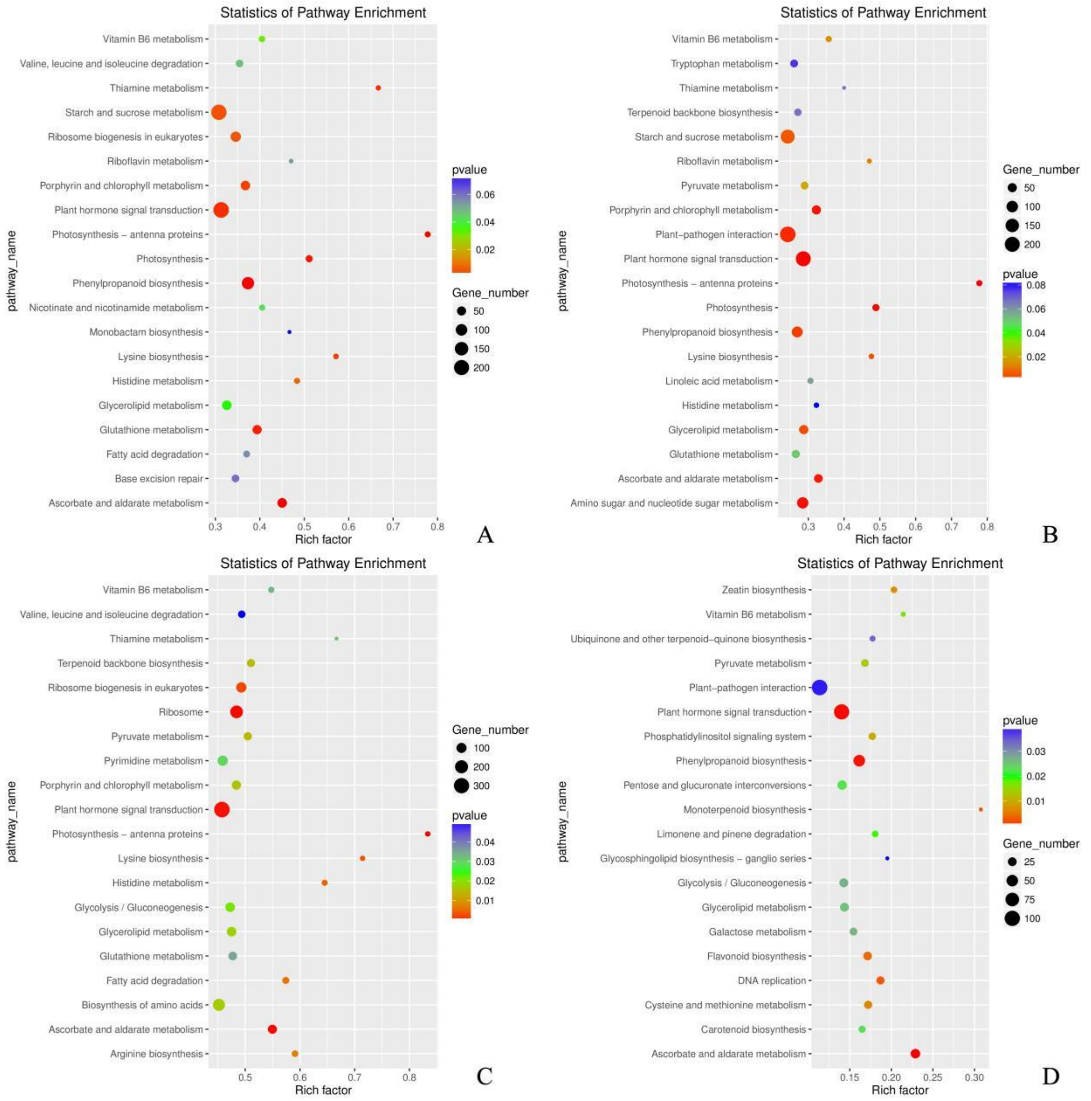

Figure S1. Top 20 KEGG significant enrichment pathways on the differently expressed genes during flower development in pink-flowered strawberry. **(A)** KEGG significant enrichment pathways in PF_L vs PF_Z; **(B)** KEGG significant enrichment pathways in PF_Z vs PF_D; **(C)** KEGG significant enrichment pathways in PF_L vs PF_D; **(D)** KEGG significant enrichment pathways in PF_L vs PF_Z vs PF_D

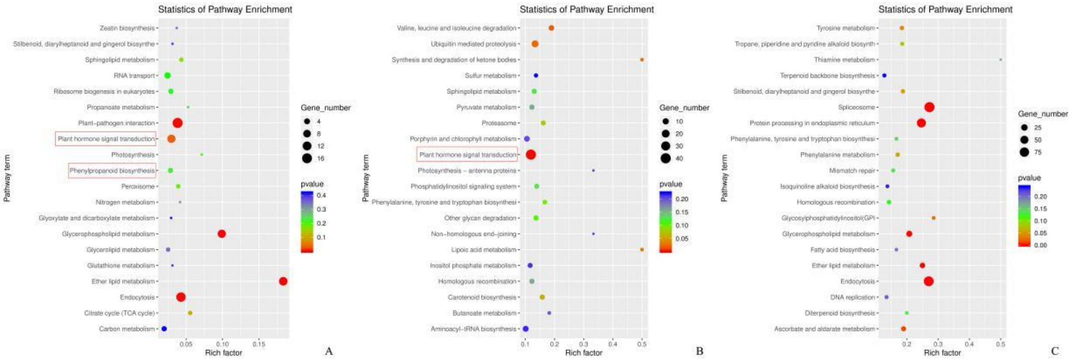

Figure S2. Top 20 KEGG significant enrichment pathways on the corresponding target genes of 3 miRNA groups that were significantly different trend (profile 1, 6, 8).

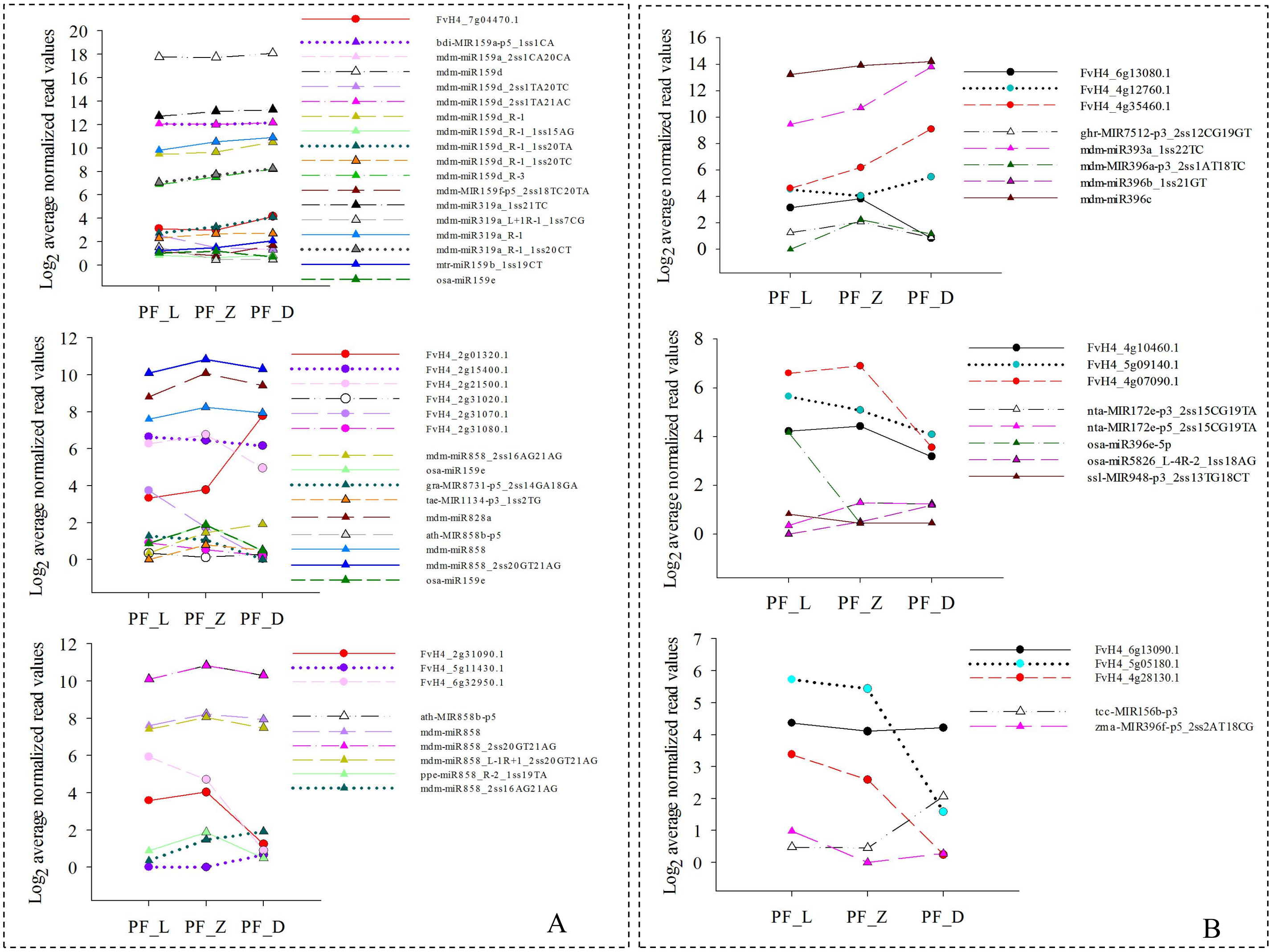
Log2 average normalized read values
PF_L
PF_Z
PF_D
FvH4_7g04470.1
bdi-MIR159a-p5_1ss1CA
mdm-miR159a_2ss1CA20CA
mdm-miR159d
mdm-miR159d_2ss1TA20TC
mdm-miR159d_2ss1TA21AC
mdm-miR159d_R-1
mdm-miR159d_R-1_1ss15AG
mdm-miR159d_R-1_1ss20TA
mdm-miR159d_R-1_1ss20TC
mdm-miR159d_R-3
mdm-MIR159f-p5_2ss18TC20TA
mdm-miR319a_1ss21TC
mdm-miR319a_L+1R-1_1ss7CG
mdm-miR319a_R-1
mdm-miR319a_R-1_1ss20CT
mtr-miR159b_1ss19CT
osa-miR159e
FvH4_2g01320.1
FvH4_2g15400.1
FvH4_2g21500.1
FvH4_2g31020.1
FvH4_2g31070.1
FvH4_2g31080.1
mdm-miR858_2ss16AG21AG
osa-miR159e
gra-MIR8731-p5_2ss14GA18GA
tae-MIR1134-p3_1ss2TG
mdm-miR828a
ath-MIR858b-p5
mdm-miR858
mdm-miR858_2ss20GT21AG
osa-miR159e
FvH4_2g31090.1
FvH4_5g11430.1
FvH4_6g32950.1
ath-MIR858b-p5
mdm-miR858
mdm-miR858_2ss20GT21AG
mdm-miR858_L-1R+1_2ss20GT21AG
ppe-miR858_R-2_1ss19TA
mdm-miR858_2ss16AG21AG
A
FvH4_6g13080.1
FvH4_4g12760.1
FvH4_4g35460.1
ghr-MIR7512-p3_2ss12CG19GT
mdm-miR393a_1ss22TC
mdm-MIR396a-p3_2ss1AT18TC
mdm-miR396b_1ss21GT
mdm-miR396c
FvH4_4g10460.1
FvH4_5g09140.1
FvH4_4g07090.1
nta-MIR172e-p3_2ss15CG19TA
nta-MIR172e-p5_2ss15CG19TA
osa-miR396e-5p
osa-miR5826_L-4R-2_1ss18AG
ssl-MIR948-p3_2ss13TG18CT
FvH4_6g13090.1
FvH4_5g05180.1
FvH4_4g28130.1
tcc-MIR156b-p3
zma-MIR396f-p5_2ss2AT18CG
B

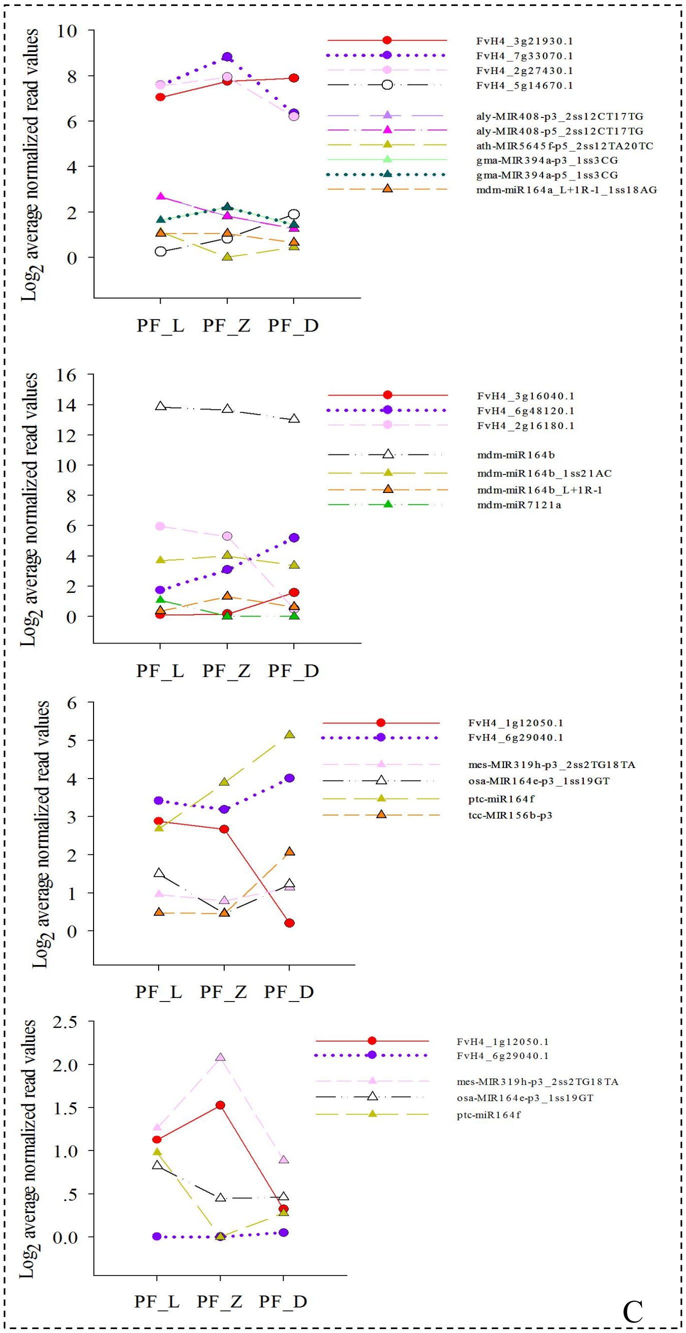

Log2 average normalized read values
PF_L PF_Z PF_D
FvH4_3g21930.1
FvH4_7g33070.1
FvH4_2g27430.1
FvH4_5g14670.1
aly-MIR408-p3_2ss12CT17TG
aly-MIR408-p5_2ss12CT17TG
ath-MIR5645f-p5_2ss12TA20TC
gma-MIR394a-p3_1ss3CG
gma-MIR394a-p5_1ss3CG
mdm-miR164a_L+1R-1_1ss18AG
FvH4_3g16040.1
FvH4_6g48120.1
FvH4_2g16180.1
mdm-miR164b
mdm-miR164b_1ss21AC
mdm-miR164b_L+1R-1
mdm-miR7121a
FvH4_1g12050.1
FvH4_6g29040.1
mes-MIR319h-p3_2ss2TG18TA
osa-MIR164e-p3_1ss19GT
ptc-miR164f
tcc-MIR156b-p3
C

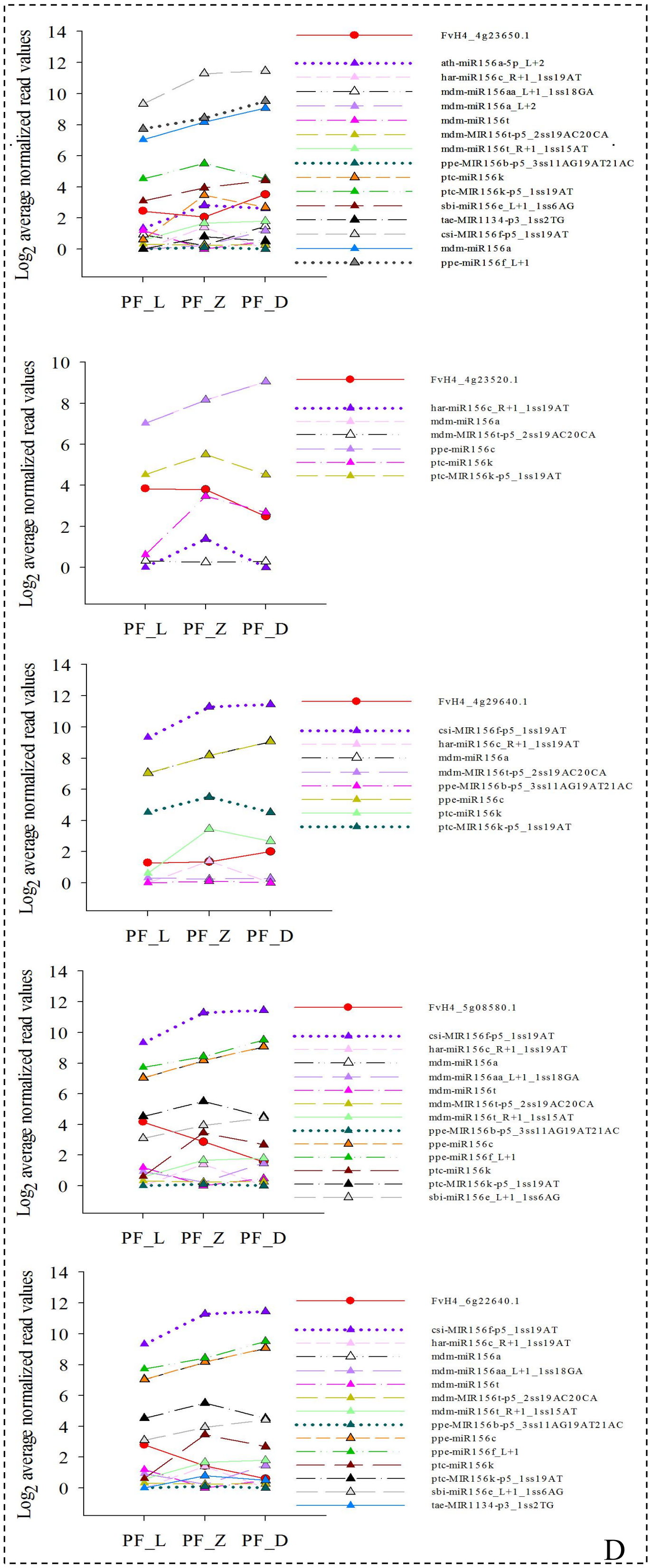

Log2 average normalized read values
PF_L PF_Z PF_D
FvH4_4g23650.1
ath-miR156a-5p_L+2
har-miR156c_R+1_1ss19AT
mdm-miR156aa_L+1_1ss18GA
mdm-miR156a_L+2
mdm-miR156t
mdm-MIR156t-p5_2ss19AC20CA
mdm-miR156t_R+1_1ss15AT
ppe-MIR156b-p5_3ss11AG19AT21AC
ptc-miR156k
ptc-MIR156k-p5_1ss19AT
sbi-miR156e_L+1_1ss6AG
tae-MIR1134-p3_1ss2TG
csi-MIR156f-p5_1ss19AT
mdm-miR156a
ppe-miR156f_L+1
FvH4_4g23520.1
ppe-miR156c
FvH4_4g29640.1
FvH4_5g08580.1
FvH4_6g22640.1
D

Figure S3. Expression profiles of selected miRNAs and targets involved in anthocyanin biosynthesis during flower development in pink-flowered strawberry. The expression profiles of the miRNAs and transcripts were obtained using the Log2 average normalized read values from sRNA-Seq and RNA-Seq analyses at PF_L, PF_Z and PF_D, respectively. **(A)** miRNAs and corresponding target *MYB* members according to the degradome sequencing. **(B)** miRNAs and corresponding target *bHLH* members according to the degradome sequencing. **(C)** miRNAs and corresponding target *NAC* members according to the degradome sequencing. **(D)** miRNAs and corresponding target *SPL* members according to the degradome sequencing. Circles and triangles represent transcripts and miRNAs, respectively.

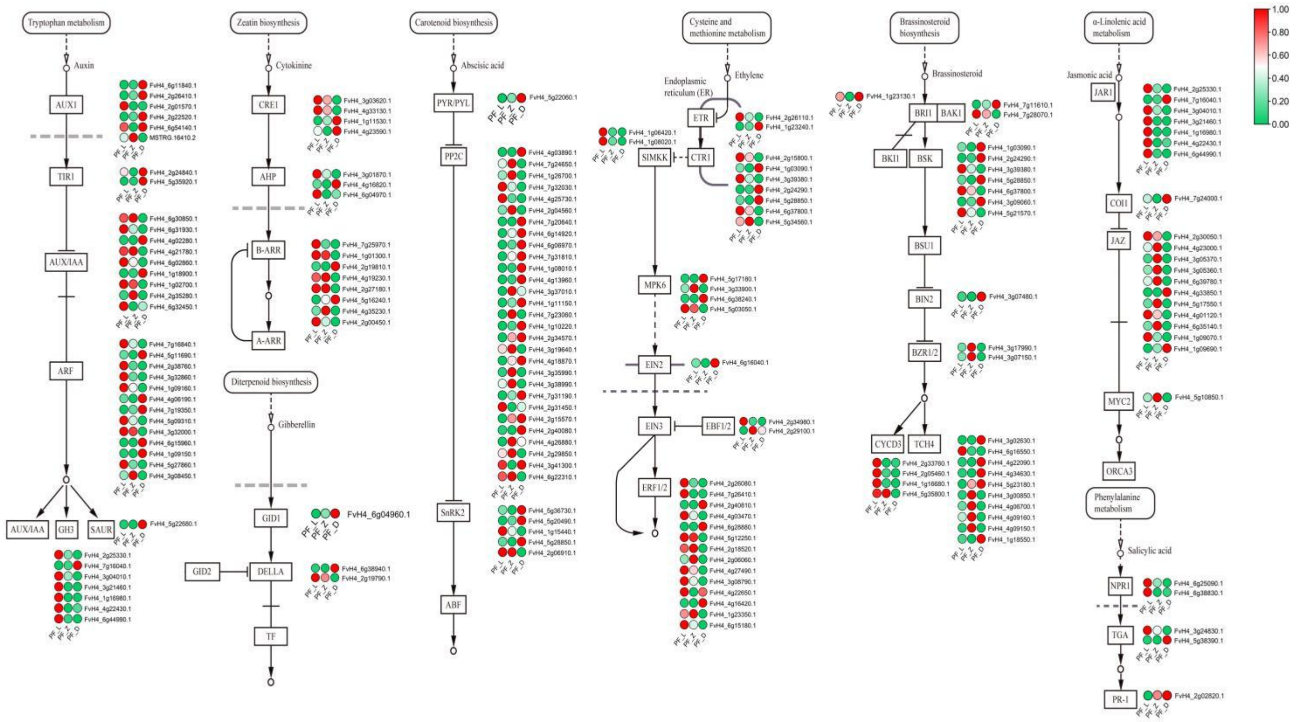

Figure S4. Heatmaps showing expression patterns of the different expression genes involved in hormone signal transduction pathways.

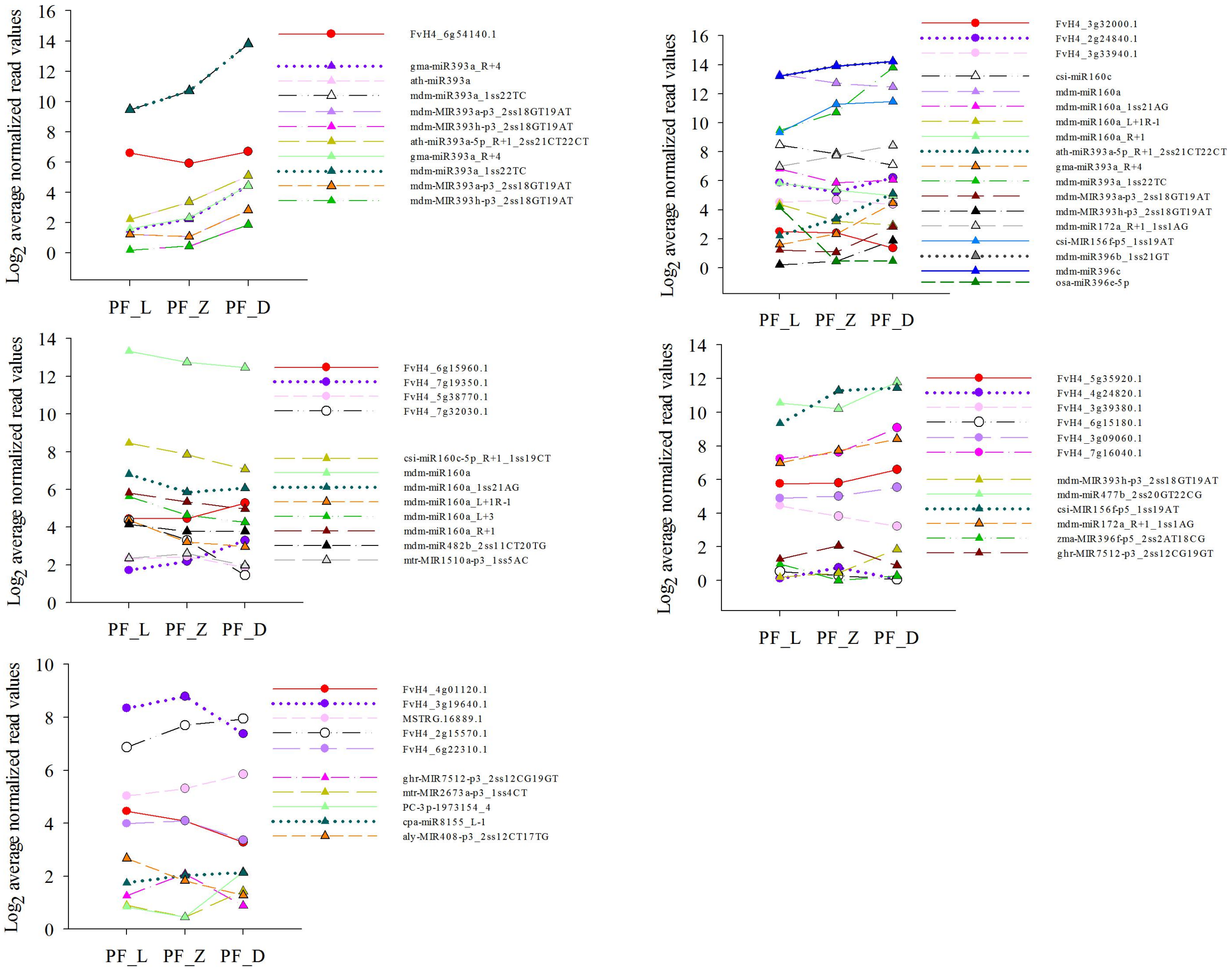

Figure S5. Expression profiles of selected miRNAs and targets involved in hormone signal transduction pathway during flower development in pink-flowered strawberry. The expression profiles of the miRNAs and transcripts were obtained using the Log_2 average normalized read values from sRNA-Seq and RNA-Seq analyses at PF_L, PF_Z, PF_D, respectively. Circles and triangles represent transcripts and miRNAs, respectively.

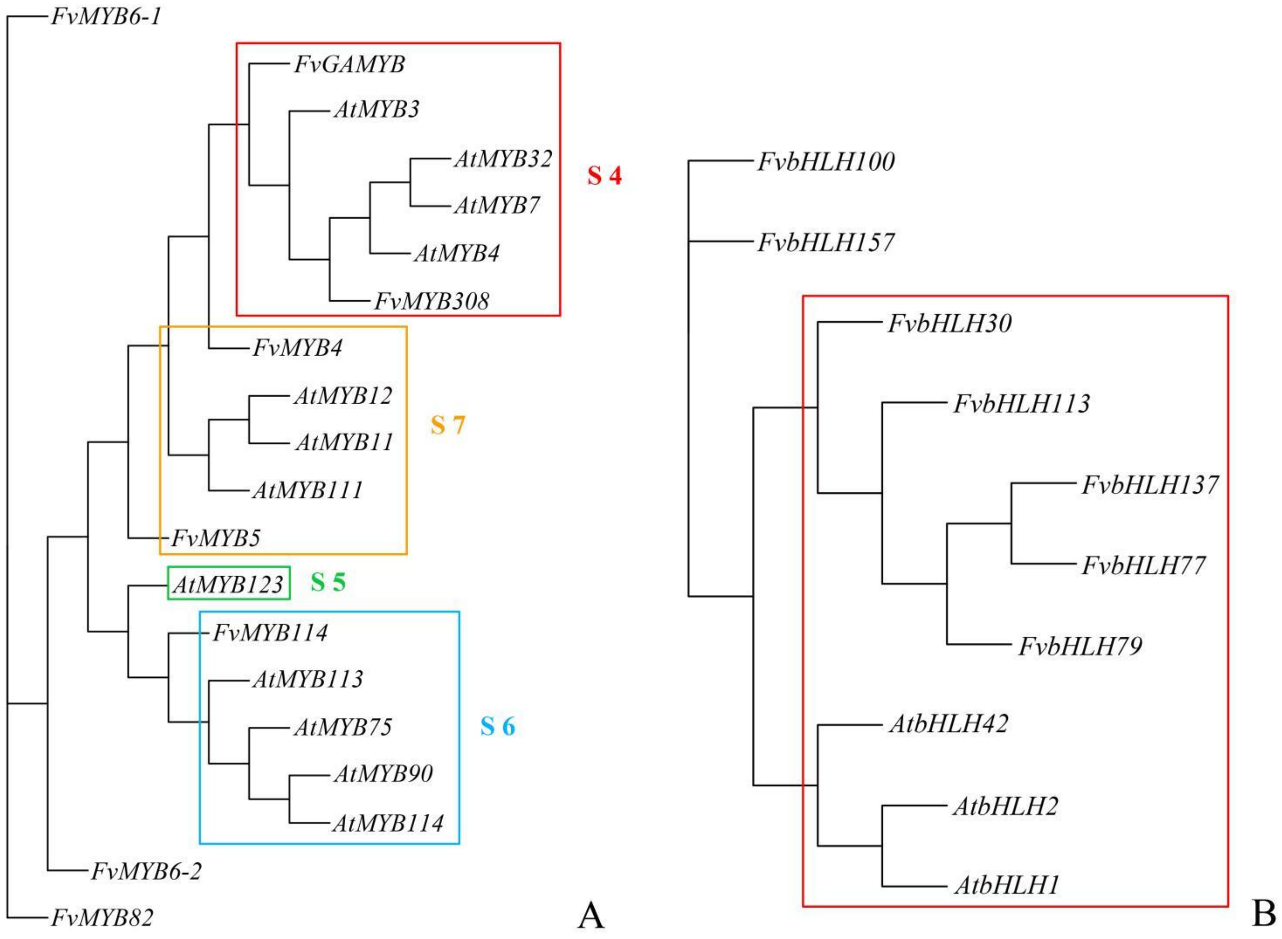

Figure S6. Phylogenetic relationships of FvMYB proteins **(A)** and FvbHLH **(B)** between F. vesca and Arabidopsis. The ML phylogenetic tree was generated using JTT algorithm with 1,000 bootstrap value via MEGA 7.0.
